# Systematic review and meta-analysis of the effects of menopause hormone therapy on cognition

**DOI:** 10.3389/fendo.2024.1350318

**Published:** 2024-03-04

**Authors:** Caroline Andy, Matilde Nerattini, Steven Jett, Caroline Carlton, Camila Zarate, Camila Boneu, Francesca Fauci, Trisha Ajila, Michael Battista, Silky Pahlajani, Paul Christos, Matthew E. Fink, Schantel Williams, Roberta Diaz Brinton, Lisa Mosconi

**Affiliations:** ^1^ Department of Population Health Sciences, Weill Cornell Medicine, New York, NY, United States; ^2^ Department of Neurology, Weill Cornell Medicine, New York, NY, United States; ^3^ Department of Radiology, Weill Cornell Medicine, New York, NY, United States; ^4^ Department of Neurology and Pharmacology, University of Arizona, Tucson, AZ, United States

**Keywords:** HRT (hormone replacement therapy), Alzheimer disease, cognition, meta-analysis, menopause

## Abstract

**Introduction:**

Despite evidence from preclinical studies suggesting estrogen’s neuroprotective effects, the use of menopausal hormone therapy (MHT) to support cognitive function remains controversial.

**Methods:**

We used random-effect meta-analysis and multi-level meta-regression to derive pooled standardized mean difference (SMD) and 95% confidence intervals (C.I.) from 34 randomized controlled trials, including 14,914 treated and 12,679 placebo participants.

**Results:**

Associations between MHT and cognitive function in some domains and tests of interest varied by formulation and treatment timing. While MHT had no overall effects on cognitive domain scores, treatment for surgical menopause, mostly estrogen-only therapy, improved global cognition (SMD=1.575, 95% CI 0.228, 2.921; *P*=0.043) compared to placebo. When initiated specifically in midlife or close to menopause onset, estrogen therapy was associated with improved verbal memory (SMD=0.394, 95% CI 0.014, 0.774; *P*=0.046), while late-life initiation had no effects. Overall, estrogen-progestogen therapy for spontaneous menopause was associated with a decline in Mini Mental State Exam (MMSE) scores as compared to placebo, with most studies administering treatment in a late-life population (SMD=-1.853, 95% CI -2.974, -0.733; *P* = 0.030). In analysis of timing of initiation, estrogen-progestogen therapy had no significant effects in midlife but was associated with improved verbal memory in late-life (*P* = 0.049). Duration of treatment >1 year was associated with worsening in visual memory as compared to shorter duration. Analysis of individual cognitive tests yielded more variable results of positive and negative effects associated with MHT.

**Discussion:**

These findings suggest time-dependent effects of MHT on certain aspects of cognition, with variations based on formulation and timing of initiation, underscoring the need for further research with larger samples and more homogeneous study designs.

## Introduction

Alzheimer’s disease (AD) is the most common cause of dementia in Western societies, with twice as many women suffering from the disorder as men (1). Postmenopausal women account for over 60% of AD patients (2, 3), a gender disparity that is not solely explained by longevity factors (4–9). Therefore, development of gender-specific AD preventative strategies is vital to combat the impending epidemic.

The discovery that the pathological process of AD may begin decades before symptom onset indicates a substantial window of time during which we may be able to intervene to halt or delay disease progression (10). This prodromal phase may start as early as midlife, thus coinciding with the menopause transition in women (11–16). There is strong biological plausibility linking menopause with AD, as estrogen, particularly 17β-estradiol, is known to have neurotrophic and neuroprotective effects (17–21), and modulatory effects on metabolic and biochemical pathways implicated in AD (9, 19, 22–25). The onset of menopause is characterized by decreased production and activity of estradiol in both the body and the brain (18), with the subsequent establishment of a hypoestrogenic state, which has been proposed as a female-specific risk factor for AD (19, 22, 24–26).

Although primarily viewed as a reproductive event, the menopause transition (perimenopause) is a neuroendocrine event associated with several neurological symptoms stemming from disruptions in estrogen-regulated thermoregulation, circadian rhythms and sensory processing, mood and cognitive functions (19). Many of these changes are risk factors for AD in turn (9, 19, 27). In particular, the fact that over 60% of menopausal women report memory changes (28–30) is concerning as memory declines, especially in verbal memory, are among the earliest neuropsychological indicators of AD (31–34).

Several studies sought to examine where estrogen supplementation by means of menopause hormone therapy (MHT) is protective against cognitive decline and AD. This hypothesis arose in large part from observational studies and small-scale clinical trials showing that MHT users had better cognitive function, especially verbal memory (35, 36), as well as a lower risk of AD or dementia than non-users (37–47). However, larger randomized controlled trials (RCTs), such as the Women’s Health Initiative Memory Study (WHIMS), reported an increased risk of all-cause dementia with estrogen-plus-progestogen therapy (EPT), and a non-significant risk increase with estrogen-only therapy (ET) for postmenopausal women ages 60 and older (48, 49). Additionally, EPT worsened cognition among older women, whereas ET had neutral effects (50).

To reconcile this discrepancy, it has been hypothesized that there may be a critical perimenopausal and early postmenopausal window or period during which estrogen exerts neuroprotection (51–55), within the context of a “healthy cell bias” of estrogen action (22). However, results from RCTs of younger menopausal women have also reported contrasting findings. Large-scale trials, such as the Women’s Health Initiative Memory Study of younger women (WHIMS-Y), the Kronos Early Estrogen Prevention Cognitive and Affective Ancillary Study (KEEPS-cog), and the Early versus Late Intervention Trial with Estradiol-Cognitive Endpoints (ELITE-cog), predominantly indicated neutral effects of MHT on cognitive function (56–59). On the other hand, several smaller studies reported positive effects of MHT on memory function (60–66) and, to some extent, verbal fluency (67).

As most studies included different populations, treatment types, and methodologies, meta-analytical examination of existing literature is warranted to systematically consolidate data from multiple sources. Herein, we conducted an updated systematic review and meta-analysis of data linking MHT to cognitive performance, encompassing findings from 34 randomized, placebo-controlled trials including 14,914 treated and 12,679 placebo participants. We examined cognitive outcomes as continuous measures; employed multi-level meta-regression analysis to examine sources of heterogeneity; and considered from the outset the impact of variables such as formulation, timing of initiation, type of menopause, and treatment duration.

## Methods

### Search criteria

In compliance with the Systematic Reviewers and Meta-Analysis (PRISMA) guidelines (68), we carried out a systematic literature search in PubMed/MEDLINE, Web of Science, and Cochrane databases from 1975 through September 2023. Key words included [‘hormone replacement therapy’, ‘estrogen therapy’, ‘estrogen replacement therapy’, ‘postmenopausal hormone therapy’] and [‘cognition’, ‘cognitive performance’, ‘memory’, ‘dementia’, ‘Alzheimer’s disease’] and [‘randomized controlled trial’, ‘clinical trial’]. This search was supplemented by a manual search of bibliographies from selected articles, reviews, and previous meta-analyses. Screening of studies was conducted by three independent authors (MN, SJ, LM). Any discrepancies were resolved by the senior author (LM).

#### Inclusion criteria

We selected only publications in the English language which met the following inclusion criteria: 1) the study was published in a peer-reviewed journal; 2) the cohort was well defined; 3) participants included medically healthy women (no comorbidities such as cardiovascular disease, stroke, or dementia) or patients with a hysterectomy/oophorectomy; 4) outcomes included measures of cognitive function; 5) studies were randomized placebo-controlled trials with at least 2 weeks duration (clinical trials without a placebo group, case reports, review papers, editorials, letters to the editor, personal communications, preclinical studies and *in vitro* research were excluded); 6) treatment was systemic estrogen with or without progestogen (studies of vaginal estradiol, tibolone, progesterone/progestin without estrogen, testosterone, and mixed preparations were excluded); 7) the outcomes were MHT vs. placebo; 8) included studies reported at least one estimate of association and one corresponding measure of statistical uncertainty such as P value, standard error, confidence interval, or data required for derivation of these estimates.

#### Data extraction

For each study, we extracted the following information: year of publication, country, study design, number of participants, participants’ ages, cognitive endpoints, treatment characteristics (e.g., timing of use, duration of use, route of administration, formulation, dosage), covariates, and summary estimates, e.g. mean or median at baseline and follow-up or change from baseline, with associated standard error, standard deviation or 95% confidence intervals (C.I.). The fully adjusted models were primarily used for analysis.

### Statistical analysis

Statistical analyses were performed using R 4.2.2 statistical software (R Core Team). To fully capitalize on the available data from the included studies, we conducted a quantitative analysis of both cognitive domains and individual cognitive tests. In sensitivity analysis, we additionally grouped study findings on the basis of selected characteristics included in **Table 1**.

**Table 1:** Randomized controlled clinical trials investigating effects of menopausal hormone therapy on cognition.

Study	Country	Design	Duration	N (treated vs. placebo)	Menopause type	Age, mean or range, years	Time since menopause	Menopausal symptoms	Therapy	Cognitive tests
(60)	Canada	Randomized, double-blind, parallel groups	2 months	19 (10 vs. 9)	Surgical	48(5)	Immediately after surgery	Unclear	Intramuscular estradiol dienanthate and estradiol benzoate, 10 mg/month, or placebo	WMS logical memory (immediate, delayed), visual reproduction, paired associates (immediate, delayed), WAIS digit span forward
(69)	Germany	Randomized, double-blind, parallel groups	2 weeks	28 (21 vs. 17)	Spontaneous and surgical	69(6)	Average 17 years	NR	Transdermal estradiol, 0.1 mg/d, or placebo	Paired associates (immediate, delayed), spatial memory, fluency (2-letter test), Stroop
(61)	UK	Randomized, double-blind, parallel groups	3 weeks	37 (19 vs. 18)	Spontaneous and surgical	55-75	>6 years	N	Transdermal 17β-estradiol 0.1 mg/d or placebo	BETAM-M, CANTAB paired associates, Stroop, Mental rotation test, Tower of London, Random Number Generation Task
(70)	USA	Randomized, double-blind, parallel groups	9 months	52 (34 vs. 18)	Spontaneous and surgical	75-91	Average 34 years	NR	Oral CEE, 0.625 mg/d +/- MPA, 5 mg/d, for 13 days every third month	WMS paired associates (immediate and delayed), Animal Test, TMT A and B, Cancellation Random Letter and Random Form Tests
(71)	Austria	Randomized, double-blind, triple groups	2 months	49 (33 vs. 16)	Spontaneous and surgical	46–67	Average 6 years	Y (Kupperman index > 15)	Oral estradiol valerate, 2 mg/d +/- dienogest, 3 mg/d, or placebo	Grünberger Verbal Memory Test, BVRT (correct reproduction and errors), Mehrfachwahl-Wortschatz-Test
(72)	USA	Randomized, double-blind, parallel groups	4-year follow-up of WHIMS study	4381 (2145 vs. 2236)	Spontaneous	65-79 years at baseline	>1 year	6% vasomotor sympotms	Oral CEE, 0.625 mg/d +/-MPA, 2.5 mg/d, or placebo	3MSE
(73)	USA	Randomized, double-blind, parallel groups	5-year follow-up of WHIMS study	2808 (1387 vs. 1421)	Spontaneous and surgical	65-79	NR	10% vasomotor symptoms	Oral CEE, 0.625 mg/d + MPA, 2.5 mg/d, or placebo	3MSE
(74)	USA	Randomized, double-blind, parallel groups	10 weeks	17 (9 vs. 8)	Spontaneous and surgical	57(7)	Average 8 years	NR	Transdermal estradiol, 0.1 mg/d, or placebo	WAIS-R logical memory (immediate, delayed), paired associates (immediate, delayed), visual reproduction (immediate, delayed) DSST and digit span total, CVLT (short and long delayed recall, recognition) Stroop test, COWAT FAS, WCST, Rey-Osterreith figure (delayed)
(75)	USA	Randomized, double-blind, parallel groups	3 years	373 (187 vs. 185)	Spontaneous and surgical	65-90	NR	NR	Oral CEE, 0.625 mg/d + MPA, 2.5 mg/d, or placebo	Digit span forward and backward, DSST, 3-Min Reasoning Test, Paired Associate Learning Test, Logical Memory, Free Recall of Words
(76)	UK	Randomized, double-blind, placebo controlled, crossover	3 months	19 (10 vs. 9)	Surgical	62-89	NR	NR	Transdermal estradiol (Femseven), 50 µg/d, or placebo	CDR (simple and choice reaction time, immediate and delayed word recall, digit vigilance, visual tracking, spatial working memory, word, picture and face recognition)
(77)	Germany	Randomized, double-blind, parallel groups	24 weeks	64 (26 vs. 38)	Spontaneous and surgical	58-75	>10 years	NR	Oral estradiol valerate, 2 mg/d, +/- progesterone, 100 mg, or placebo	Rivermead Behavioral Memory Test paragraph recall (immediate, delayed), Verbal paired associates, Visual paired associates, Digit span forward and backward, Stroop test, Verbal fluency (letter and category), Mental Rotation Test, Timed cancellation task
(78)	Australia	Randomized, double-blind, parallel groups	24 weeks	115 (58 vs. 57)	Surgical	74(4)	29 years	12-20% with vasomotor symptoms	Oral estradiol, 0.5-1-2 mg/d, or placebo	CAMCOG, FAS + Animals, Block Design test, CVLT-II (total, delayed recall), Test Faces
(63)	USA	Randomized, double-blind, parallel groups	3 months	50 (26 vs. 24)	Spontaneous and surgical	51(4)	NR	62% with symptoms (30% mild, 14% moderate, 18% severe)	Transdermal 17β-estradiol, 0.05 mg/day, or placebo	CVLT (short and long delayed recall), WMS-R delayed memory and visuospatial memory, digit span total, Rey-Osterreith Complex Figure Test (short and long term)
(79)	USA	Randomized, double-blind, parallel groups	3 years	1416 (693 vs. 723)	Spontaneous	74(4)	Average 22 years	5% with moderate to severe HF	Oral CEE, 0.625 mg/d + MPA, 2.5mg, or placebo	PMA-Vocabulary, FAS + Animals, BVRT, CVLT (short and long delay free recall), Digit span forward and backward, Card rotations
(80)	USA	Randomized, triple-blind, parallel groups	2 years	417 (208 vs. 209)	Spontaneous	60-80	>5 years	Y (16% with VSM)	Ultralow-dose transdermal 17β-estradiol, 0.014 mg, or placebo + calcium and vitamin D	3MS, Logical Memory (immediate, delayed), Brief Visuospatial Memory Test, Word List Memory, TMT-B, Modified Boston Naming, Verbal Fluency (Category Fluency)
(64)	USA	Randomized, double-blind, parallel groups	2 months	32 (14 vs. 18)	Spontaneous and surgical	53(1)	3-36 months	Y (average 4 hot flashes daily)	Oral estradiol, 2mg/d, or placebo	WMS paragraph recall (immediate, delayed); paired associates (immediate, delayed); visual reproduction (immediate, delayed); FAS
(81)	USA	Randomized, double-blind, parallel groups	4 months	158 (77 vs. 81)	Spontaneous	44-62	1-2 years	Y (50% symptomatic)	Oral CEE, 0.652 mg/d +/- MPA, 2.5 mg/d, or placebo	CLVT (learning, short delay recall, long delay recall, long delay recognition), BVRT, Logical memory (immediate, delayed), digit span forward and backward, brief test of attention, FAS, Card rotations
(82)	USA	Randomized, double-blind, parallel groups	3 years	57 (32 vs. 27)	Spontaneous and surgical	76(6)	>5 years	NR	Transdermal 17β-estradiol, 0.25 mg/d +/- micronized progesterone, 100 mg	COWAT FAS + animals, TMT-B, Wisconsin Card Sorting Task, Boston Naming, Digit Symbol Modalities Test, Rey-Osterreith Complex Figure (immediate, delayed), WMS-III Paired Associates
(83)	Brazil	Randomized, double-blind, parallel groups	3 months	65 (34 vs. 31)	Spontaneous	48-65	66(53) months	Y ([ … ] decrease in the total score of the Greene Climacteric Scale)	Oral 17β-estradiol, 2 mg/d, or placebo	MMSE, Digit span, evocation of story, Bells Test, DSST, Stroop Test, TMT A and B, FAS
(67)	USA	Randomized, double-blind, parallel groups	12 months	18 (9 vs. 9)	Spontaneous	44-62	10months to 6 years	Y (≥35 hot flushes per week)	Oral CEE, 0.652 mg/d +/- MPA, 2.5 mg/d, or placebo	CVLT-M, logical memory, BVRT, digit span forward and backward, FAS, brief test of attention, Card rotation task
(84)	USA	Randomized, double-blind, parallel groups	3 years+3 year follow-up	886 (434 vs. 452)	Surgical	74(4)	NR	10% moderate to severe VMS	Oral CEE 0.625mg/d or placebo	PMA-Vocabulary, FAS + Categories, CVLT, WAIS-R digit span forward and backward, Card Rotations test
(85)	Canada	Randomized, double-blind, parallel groups	2 years	142 (70 vs. 72)	Spontaneous and surgical	61-87	37-51 months	Y (various)	Oral 17β-estradiol, 1mg/d + norethindrone, 0.35 mg, or placebo	CVLT (immediate recall, new list recall, cued recall and recognition memory)
(86)	Finland	Randomized, double-blind, parallel groups	6 months	16 (8 vs. 8)	Spontaneous	63	12(5) years	Y (Modified Kupperman Index, night sweating and hot flushes, scale 2–8 points)	Oral estradiol valerate, 2 mg/d + norethisterone, 0.7 mg, or placebo	CogniSpeed software, Digit span total, PASAT, WAIS-R similarities, RAVLT immediate and delayed, DSST, Block Design test, BVRT (immediate and delayed), Stroop
(50)	USA	Randomized, double-blind, parallel groups	5-year follow-up of WHIMS study	2808 (1387 vs. 1421)	Spontaneous and surgical	65-79	NR	10% vasomotor symptoms	Oral CEE, 0.625 mg/d + MPA, 2.5 mg/d, or placebo	3MSE, PMA-Vocabulary, FAS + animal naming, BVRT, CVLT-A (long and short delayed recall), digit span forward, backward, and total; Card Rotation Test
(65)	Brazil	Randomized, double-blind, parallel groups	6 months	53 (26 vs. 27)	Surgical	43-56	Average 3 years	Y (Blatt-Kupperman Index and the Menopause Rating Scale)	Oral CEE, 0.625 mg/d, or placebo	Digit span forward and backward, DSST, WMS-R Paired associates (total), Logical Memory (immediate, delayed), Free Recall of Words
(87)	Sweden	Randomized, double-blind, parallel groups	4 weeks	200 (134 vs. 66)	Spontaneous and surgical	50-65	9(4) years	NR	Oral estradiol valerate, 2mg/d, or placebo	Composite verbal memory, visual memory and verbal fluency scores
(88)	Germany	Randomized, double-blind, parallel groups	6 months	23 (12 vs. 11)	Spontaneous	53	Average 2 years	NR	Oral estradiol, 1 mg + drospirenone, 2mg, or placebo	CogState visual paired associate (learning), International Shopping list (learning and recall), Groton Maze (learning and recall)
(56)	USA	Randomized, double-blind, parallel groups	14-year follow-up of WHIMS study	1168 (609 vs. 559)	Spontaneous and surgical	68	Average 20 years	NR	Oral CEE 0.625 mg/d + 2.5 mg/d MPA or placebo	TICS-m; EBMT (immediate, delayed); digit span forward, backward, total; VF-A; TMT A and B
(89)	USA	Randomized, double-blind, parallel groups	12 week	29 (16 vs. 13)	Spontaneous and surgical	45-55	Average 18 months	NR	Oral estradiol, 1 mg, +/- progesterone, 200 mg, or placebo	HVLT-R (total, delayed), BVMT-R (delayed)
(58)	USA	Randomized, double-blind, parallel groups	2.5 and 5-year	643 (323 vs. 320)	Spontaneous and surgical	55(5)	¾6 or >10	NR	Oral 17β-estradiol, 1 mg/d +/- cyclic micronized progesterone, 45 mg, or placebo	Composite verbal memory, global cognition and executive function scores
(59)	USA	Randomized, double-blind, parallel groups	5 and 7-year follow-up of WHIMS study	4216 (2153 vs. 2063)	Spontaneous and surgical	50-54 and 65-79	NR	NR	Oral CEE, 0.625 mg/d + MPA, 2.5 mg/d, or placebo	TICS-m, EBMT (immediate, delayed), OTMT, VF-A, Digit Span forward, backward, total
(66)	USA	Randomized, double-blind, parallel groups	4 months	136 (68 vs. 68)	Spontaneous	50-60	1-10	Y (Greene climacteric scales were negatively correlated after the intervention)	Oral CEE, 0.625mg/d + MPA, 2.5mg/d, plus 500 mg calcium+200 IU vitamin D	MoCA
(90)	USA	Randomized, double-blind, parallel groups	4 years	67 (38 vs. 29)	Spontaneous and surgical	42 – 58	8	NR	Oral CEE, 0.45mg/d, or transdermal 17β-estradiol 50µg/d + oral progesterone, 200mg/d for 12 days, or placebo	Composite global cognition score

BVRT, Benton Visual Retention Test; CAMCOG, Cambridge Cognitive Examination for Mental Disorders of the Elderly; CANTAB, Cambridge Neuropsychological Test Automated Battery; CDR, Cognitive Drug Research computerized assessment; COWAT, Controlled Oral Word Association Test; CVLT, California Verbal Learning Test; CEE, conjugated equine estrogen; DSST, Digit Symbol Substitution Test; EBTM, East Boston Memory Test; HVLT, Hopkins Verbal Learning test; MoCA, Montreal Cognitive Assessment; MMSE, Mini Mental State Examination; MPA, medroxyprogesterone acetate; NR, not reported; OTMT, Oral Trail Making Test; PASAT, Paced Auditory Serial Addition test; PMA, primary mental abilities; RAVLT, Rey Auditory Verbal Learning Test; TICS-m, Telephone Interview for Cognitive Status-modified; TMT, Trail Making Test; VF-A, Verbal Fluency-Animals; WAIS, Wechsler Adult Intelligence Scale; WMS, Wechsler Memory Scale.

#### Meta-analysis with Robust Variance Estimation

Only RCTs that reported mean outcome scores at baseline and follow-up, raw or standardized mean change, and/or standardized mean difference with corresponding standard error, standard deviation, or 95% C.I, were included in analysis. Meta-analysis was performed for cognitive domains and individual tests available across at least four studies reporting comparable outcomes and exposure groupings (91, 92). As several studies include multiple effect estimates for different exposure types, as well as multiple outcome measures, we applied Robust Variance Estimation (RVE) to compute the pooled effect size (93). This method accounts for intra-study dependent effect sizes while mitigating the impact of outliers, unequal variances, and other sources of heterogeneity (93). We evaluated the presence of heterogeneity using Cochran’s Q, I^2^ and tau^2^ statistics (94). Because different study designs and outcome measures were used across studies, we interpreted data using random-effect models, which use a weighting scheme that incorporates study sample size and within- and between-study variance to account for study heterogeneity (95–97).

Study-specific estimates were used to calculate pooled estimated effect sizes (standardized mean difference [SMD]) with 95% C.I. Results were considered significant at *P* < 0.05. When interpreting SMDs, the magnitude is a measure of effect size: a SMD of approximately 0.2 represents a small effect size, e.g. a small difference between the groups. A SMD of approximately 0.5 represents a medium effect size, e.g. a moderate difference between the groups. A SMD of 0.8 or greater indicates a large effect size, e.g. a substantial difference between the groups.

Our primary analysis focused on cognitive categories or domains, defined as different tests measuring the same cognitive ability which are first standardized and then analyzed as a group. As done in previous Cochrane and other meta-analyses (36, 98–101), we grouped together assessments evaluating the same type of cognitive function into separate domains: verbal memory [e.g., paragraph recall/logical memory, paired associates, California Verbal Learning Test (CVLT)]; working memory [e.g., digit symbol substitution (DSST), digit span forward and backward], visuospatial memory [e.g. Benton Visual Retention Test (BVRT), Rey Visual Learning Test (RVLT)], verbal fluency (e.g., COWAT, animal naming, FAS), executive function (e.g. Stroop, Trail Making Test, TMT-B], visuospatial abilities [e.g. mental rotation, card sorting test], and global cognition [e.g., Mini Mental State Exam (MMSE), Montreal cognitive Assessment (MoCA), Cambridge Cognitive Examination for Mental Disorders of the Elderly (CAMCOG)]. The full list of tests included in each domain is found in **Supplementary Table 1**. For tests exhibiting an inverse association with performance (e.g. Stroop, TMT-B), the sign of the calculated standardized mean difference was inverted before grouping. As different studies categorized certain tests differently, we based our grouping on generally accepted criteria and insights from previous meta-analyses and Cochrane reviews. The process of grouping tests into domains was a collaborative effort involving three authors (MN, SJ, CC).

Secondarily, we tested whether specific cognitive tests are more sensitive to MHT effects than others, with a focus on those known to have estrogen-sensitive effects (79, 81, 102). Our primary outcomes of interest were as follows: short- and long-delayed recall assessed using the CVLT; immediate and delayed recall of paired associates; FAS; TMT-B; digit span forward and backward. We also examined MMSE and digit span total as enough studies reported these estimates.

We then conducted a sensitivity analysis testing for effects of MHT formulation (ET, EPT), initiation timing (midlife or age ¾65 years, late-life or age >65 years), menopause type (surgical, spontaneous), and treatment duration (¾1 year, >1 year). Analyses were repeated after excluding data from post-intervention studies (50, 59), as only ~4% of participants reported using hormone therapy after the trials termination (103).

#### Multi-level meta-regression

To further examine and account for heterogeneity in our dataset, we employed a multi-level meta-regression analysis incorporating potential modifier variables using the ‘metafor’ package in R4.2.2. This allowed us to obtain estimates for the effect of each modifier on the pooled estimate and thus identify drivers of the observed heterogeneity. The multi-level approach allowed inclusion of multiple estimates per study, incorporating hierarchical structuring to account for induced correlations of estimates that may arise from any given study and for within- and between-study dependencies across those parameters. An unstructured variance-covariance matrix was used for random effects variances (104). Covariates included in the model were MHT formulation (ET, EPT), initiation timing (midlife, late-life), menopause type (surgical, spontaneous), treatment duration (¾1 year, >1 year), and study size (<100, 100-500, >500). Changes to effect sizes due to confounders were assessed at *P* < 0.05.

#### Examination of publication bias

For analyses including 10 or more studies, possible publication bias was evaluated using Egger’s tests and funnel plots (105). For subgroups with significant publication bias, the Duval and Tweedie Trim and Fill method was applied which imputes the effects of missing studies using a random-effect modeling framework, and then re-computes the pooled effect size (106).

## Results

### Literature search and characteristics of included studies

**Figure 1:**
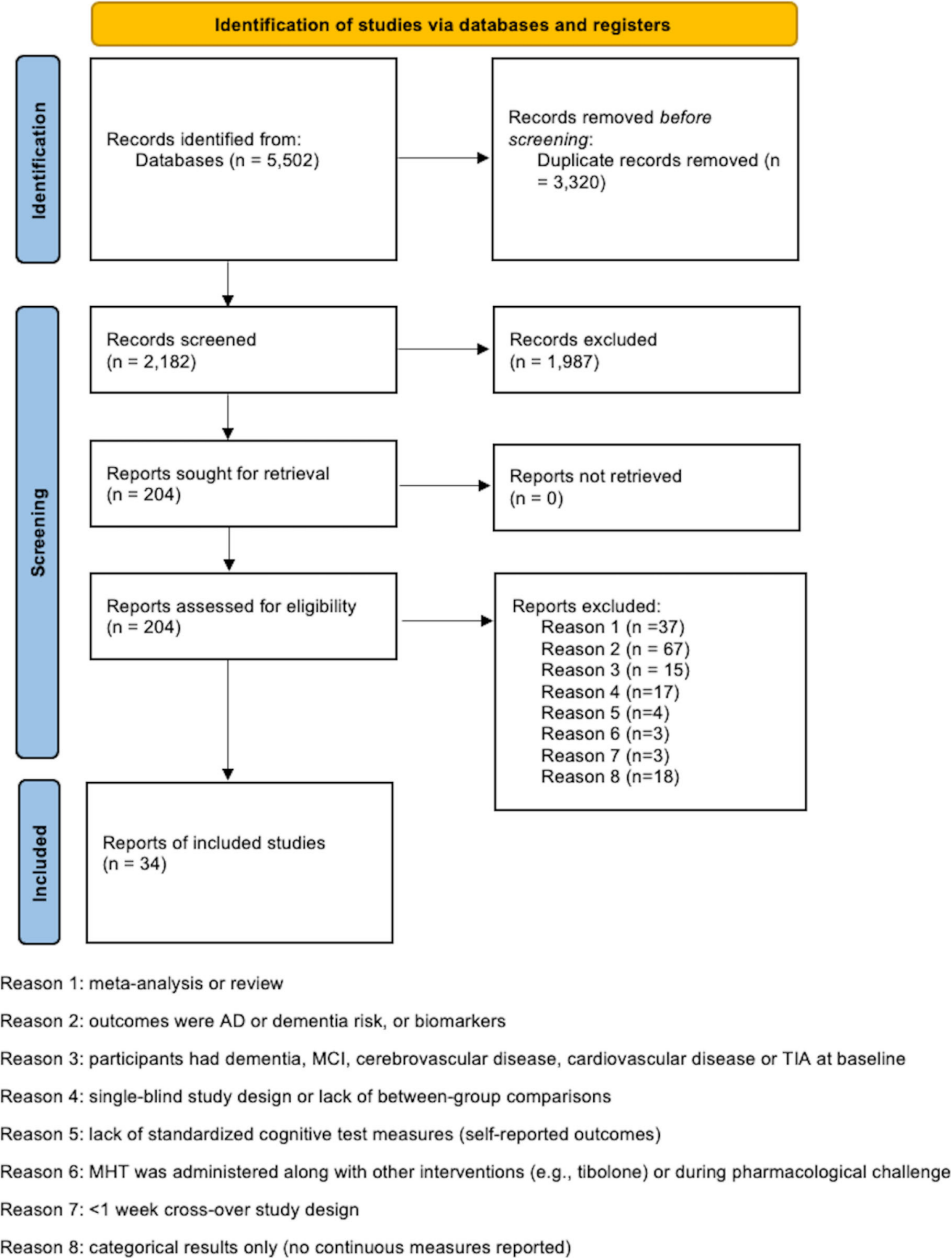
PRIMSA Flow chart.

Our PRISMA flow diagram is shown in **Figure 1**. Our systematic search initially identified 5,502 papers, of which 3,320 were found to be duplicates. From the remaining studies, 1,987 papers failed to meet the inclusion criteria and were excluded during title and/or abstract screening. The remaining 204 articles were selected for full-text inspection. Among these, a total of 34 eligible studies were pooled together for analysis.

### Demographics

Key characteristics of pooled studies are found in **Table 1**. Thirty-four studies including quantitative data were examined for associations between MHT and cognition in this meta-analysis. The final dataset included a total of 14,914 treated and 12,679 placebo participants. However, the participant distribution in these studies exhibited significant variability, with an average of 719 participants per study, but a median of only 65.

The duration of the studies also showed a wide range. On average, the studies spanned a period of 24 months, yet the median was notably shorter at 6 months. Duration of treatment was ¾1 year in 58.8% of studies, while the remaining 41.2% extended beyond 1 year.

Regarding the menopausal status of the participants, 29.4% of the studies reported data on women who had undergone menopause spontaneously, and 20.6% focused on women who had undergone hysterectomies. The remaining studies included mixed groups. Additionally, 35.3% of studies included women who were ¾6 years post-menopause, another 44.1% included women who were >6 years post-menopause, 17.6% included mixed groups, and 2.9% did not report this information.

About a third of the studies (35.3.5%) reported on MHT as their primary outcome. Of those which reported on specific formulations, 44.1% utilized ET as the only therapy, and 20.6% utilized EPT. The mode of administration was predominantly oral (73.5%), followed by transdermal applications in 20.6% of the cases, while 2.9% used either oral or transdermal methods, and 2.9% employed intramuscular bolus injections. In terms of the specific hormones used, estradiol was the primary hormone used in 55.9% of studies. Conjugated equine estrogen (CEE) was the primary hormone in 41.2% of the studies). Additionally, 2.9% of studies used either estradiol (E2) or CEE. Among estradiol studies, 35.0% were in combination with a progestogen, chiefly micronized progesterone, as well as norethindrone/norethisterone, drospirenone, and dienogest. Among CEE studies, 73.3% were in combination with medroxyprogesterone acetate (MPA).

Almost half (47.1%) of the studies did not provide information on presence of menopausal symptoms. Among those that reported these data, menopausal symptoms were absent in 1 study and present, at least for some women, in the remaining 17 studies (50.0%). However, the average frequency of menopausal symptoms was 32.5%, with a median of only 16%.

By location, the majority of RCTs were conducted in America (58.8%), followed by Northern Europe (18%), Canada (5.9%), Brazil (5.8%), Iran (2.9%) and Australia (2.9%) (**Table 1**).

### Primary analysis: meta-analysis of cognitive domains

Results are summarized in **Table 2**. Due to presence of heterogeneity across most cognitive domains (*P* ¾ 0.031), we interpreted the random effect model estimates. Pooled estimates from random-effects meta-analysis indicated no significant effects of MHT on cognitive domain scores compared to placebo (*P* ≤ 0.130; **Supplementary Figures 1-8**). Excluding the two post-intervention studies left results largely unchanged (**Table 2**). Possible sources of heterogeneity were investigated in the sensitivity analysis and meta-regression analysis, below.

**Table 2:** Effects of MHT on cognitive domain scores.

	Standardized mean difference (95% CI)	P-value	Heterogeneity p-value	Publication bias p-value
Entire dataset				
Global cognition	-0.343 (-1.239, 0.553)	0.370	<0.001	0.347
Verbal memory	-0.161 (-0.666, 0.344)	0.478	<0.001	0.507
Verbal memory, delayed	-0.277 (-0.947, 0.393)	0.373	0.001	0.655
Visual memory	-0.270 (-1.07, 0.53)	0.468	0.001	0.595
Visuospatial skills	0.364 (-1.397, 2.125)	0.615	0.031	0.541
Working memory	-0.404 (-1.471, 0.662)	0.382	0.001	0.958
Fluency	-0.356 (-0.881, 0.169)	0.130	0.001	0.707
Executive function	-0.884 (-3.349, 1.582)	0.356	0.001	0.911
Language	0.402 (-1.764, 2.569)	0.454	0.003	NA
Excluding post-intervention studies				
Global cognition	-0.369 (-1.347, 0.608)	0.377	0.001	0.417
Verbal memory	-0.177 (-0.767, 0.414)	0.509	0.001	0.509
Verbal memory, delayed	-0.277 (-0.947, 0.393)	0.373	0.001	0.655
Visual memory	-0.321 (-1.05, 0.408)	0.356	0.001	0.394
Visuospatial skills	0.414 (-1.845, 2.673)	0.646	<0.001	0.363
Working memory	-0.404 (-1.471, 0.662)	0.382	0.001	0.958
Fluency	-0.486 (-1.175, 0.203)	0.127	0.005	0.725
Executive function	-1.144 (-4.359, 2.07)	0.351	0.002	0.919
Language	0.988 (-0.329, 2.304)	0.067	<0.001	NA

Point estimates are pooled standardized mean differences (SMD) calculated through meta-analysis. SMDs and associated 95% confidence interval, CI, are reported for each cognitive domain, along with corresponding P-values. Positive SMDs indicate an improvement in the treated group compared to the placebo group, whereas negative SMDs indicate the opposite effect. Estimates are reported for the entire dataset and after exclusion of post-intervention studies (50, 59). Results are considered significant at p<0.05. Analyses with fewer than 10 studies are marked as not applicable (NA) for the publication bias p-values.

#### Effects of treatment duration

Results are found in **Table 3**. Pooled estimates from random effects meta-analysis indicated no changes in cognitive domain scores with shorter duration of MHT treatment, and a worsening in visual memory with longer treatment duration (SMD = -1.577, 95% CI -2.261, -0.892; *P* = 0.022).

**Table 3:** Effects of MHT on cognitive domains by duration of treatment.

	Standardized mean difference (95% CI)	P-value	Heterogeneity p-value	Publication bias p-value
Duration ¾1 year				
Global cognition	0.125 (-0.407, 0.657)	0.317	<0.001	NA
Verbal memory	0.062 (-1.897, 2.021)	0.939	0.091	0.740
Verbal memory, delayed	-0.382 (-1.675, 0.91)	0.500	0.045	0.713
Visual memory	0.163 (-0.536, 0.861)	0.605	0.015	0.456
Visuospatial skills	-0.469 (-3.4, 2.462)	0.580	<0.001	NA
Working memory	-2.272 (-8.656, 4.111)	0.158	0.034	NA
Fluency	-0.929 (-4.715, 2.857)	0.368	<0.001	NA
Executive function	-0.743 (-8.121, 6.634)	0.763	0.002	NA
Language	NA	NA	NA	NA
Duration >1 year				
Global cognition	0.018 (-0.343, 0.378)	0.648	<0.001	NA
Verbal memory	-0.385 (-1.276, 0.506)	0.282	0.001	NA
Verbal memory, delayed	-0.501 (-1.774, 0.773)	0.276	<0.001	NA
Visual memory	-1.577 (-2.261, -0.892)	0.022	0.001	NA
Visuospatial skills	2.509 (-3.622, 8.639)	0.121	<0.001	NA
Working memory	0.168 (-1.031, 1.367)	0.640	<0.001	NA
Fluency	-0.14 (-2.522, 2.243)	0.709	<0.001	NA
Executive function	-0.022 (-0.127, 0.083)	0.231	<0.001	NA
Language	0.988 (-0.329, 2.304)	0.067	<0.001	NA

Point estimates are pooled standardized mean differences (SMD) calculated through meta-analysis. SMDs and associated 95% confidence interval, CI, are reported each cognitive domain, along with corresponding P-values. Results are considered significant at p<0.05. Positive SMDs indicate an improvement in the treated group compared to the placebo group, whereas negative SMDs indicate the opposite effect. Estimates are reported for studies of shorter (≤1 year) and longer duration (>1year). Analyses with fewer than 10 studies are marked as not applicable (NA) for the publication bias p-values.

#### Effects of timing

Results are found in **Table 4**. Pooled estimates from random effects meta-analysis indicated no significant effects of MHT during midlife. In contrast, MHT in late-life was associated with mild reductions in global cognition (SMD = -0.071, 95% CI -0.077, -0.065; *P* = 0.004).

**Table 4:** Effects of MHT on cognitive domains by initiation timing.

	Standardized mean difference (95% CI)	P-value	Heterogeneity p-value	Publication bias p-value
Midlife or before age 65				
Global cognition	0.033 (-0.246, 0.312)	0.375	NA	NA
Verbal memory	-0.026 (-0.638, 0.585)	0.687	0.061	0.204
Verbal memory, delayed	-0.022 (-0.192, 0.147)	0.346	<0.001	0.134
Visual memory	0.248 (-0.042, 0.538)	0.06	0.105	0.336
Visuospatial skills	0.150 (-8.820, 9.120)	0.869	<0.001	NA
Working memory	-0.826 (-3.214, 1.562)	0.351	<0.001	0.435
Fluency	-0.053 (-1.009, 0.903)	0.609	<0.001	NA
Executive function	-1.98 (-25.104, 21.143)	0.493	NA	NA
Language	NA	NA	NA	NA
Late life or after age 65				
Global cognition	-0.071 (-0.077, -0.065)	0.004	<0.001	NA
Verbal memory	-0.689 (-1.754, 0.376)	0.154	0.001	0.155
Verbal memory, delayed	-0.685 (-1.66, 0.29)	0.126	<0.001	0.398
Visual memory	-0.726 (-2.337, 0.885)	0.299	0.001	0.519
Visuospatial skills	0.379 (-4.505, 5.264)	0.802	<0.001	NA
Working memory	0.121 (-1.052, 1.294)	0.721	<0.001	NA
Fluency	-0.175 (-1.755, 1.404)	0.597	<0.001	0.907
Executive function	-0.05 (-0.412, 0.311)	0.327	<0.001	NA
Language	0.988 (-0.329, 2.304)	0.067	<0.001	NA

Point estimates are pooled standardized mean differences (SMD) calculated through meta-analysis. SMDs and associated 95% confidence interval, CI, are reported each cognitive domain, along with corresponding P-values. Results are considered significant at p<0.05. Positive SMDs indicate an improvement in the treated group compared to the placebo group, whereas negative SMDs indicate the opposite effect. Estimates are reported for studies assessing MHT in midlife or before age 65 and for late-life or after age 65 years. Analyses with fewer than 10 studies are marked as not applicable (NA) for the publication bias p-values.

#### Effects of menopause type and formulation

Results are found in **Table 5**. MHT for surgical menopause, mainly ET, was associated with moderate improvements in global cognition (SMD = 1.575, 95% CI 0.228, 2.921; *P* = 0.043). MHT for spontaneous menopause had no significant effects on cognition.

**Table 5:** Effects of MHT on cognitive domains by menopause type and formulation.

	Standardized mean difference (95% CI)	P-value	Heterogeneity p-value	Publication bias p-value
Estrogen-only therapy for surgical menopause				
Global cognition	1.575 (0.228, 2.921)	0.043	<0.001	NA
Verbal memory	-0.474 (-2.288, 1.34)	0.385	0.003	0.877
Verbal memory, delayed	-0.527 (-1.808, 0.754)	0.258	<0.001	0.828
Visual memory	-1.171 (-4.787, 2.445)	0.284	0.001	NA
Visuospatial skills	NA	NA	NA	NA
Working memory	-0.133 (-2.678, 2.412)	0.882	0.005	0.743
Fluency	-0.714 (-7.21, 5.783)	0.672	0.027	NA
Executive function	1.213 (-1.069, 3.495)	0.094	<0.001	NA
Language	NA	NA	NA	NA
Estrogen-progestogen therapy for spontaneous menopause				
Global cognition	-0.974 (-3.025, 1.078)	0.106	<0.001	NA
Verbal memory	0.26 (-0.423, 0.943)	0.403	0.07	NA
Verbal memory, delayed	0.265 (-1.795, 2.324)	0.727	0.001	NA
Visual memory	-0.255 (-2.949, 2.439)	0.704	0.092	0.578
Visuospatial skills	0.933 (-1.736, 3.601)	0.151	<0.001	NA
Working memory	-0.194 (-0.786, 0.398)	0.456	0.102	NA
Fluency	1.092 (-1.334, 3.518)	0.115	0.003	0.762
Executive function	0.633 (-9.879, 11.146)	0.850	0.003	NA
Language	NA	NA	NA	NA
Estrogen-only (any type of menopause)				
Global cognition	-0.178 (-2.031, 1.675)	0.802	<0.001	NA
Verbal memory	-0.452 (-1.252, 0.347)	0.205	0.001	0.758
Verbal memory, delayed	-0.525 (-1.329, 0.279)	0.162	<0.001	0.941
Visual memory	-0.4 (-1.465, 0.664)	0.411	0.001	0.438
Visuospatial skills	0.395 (-2.515, 3.306)	0.708	<0.001	NA
Working memory	-0.134 (-2.037, 1.769)	0.858	0.005	0.755
Fluency	-0.871 (-2.662, 0.92)	0.267	0.021	0.606
Executive function	0.413 (-3.19, 4.016)	0.762	<0.001	0.431
Language	NA	NA	NA	NA
Estrogen-progestogen (any type of menopause)				
Global cognition	-1.399 (-5.569, 2.771)	0.164	<0.001	NA
Verbal memory	0.453 (-0.312, 1.219)	0.199	0.056	0.642
Verbal memory, delayed	0.065 (-2.923, 3.052)	0.944	0.001	NA
Visual memory	-1.057 (-3.345, 1.231)	0.179	0.22	NA
Visuospatial skills	-0.257 (-2.096, 1.582)	0.360	<0.001	NA
Working memory	-0.264 (-1.301, 0.774)	0.508	0.117	0.661
Fluency	1.168 (-0.176, 2.513)	0.058	0.015	NA
Executive function	-3.942 (-9.076, 1.192)	0.065	<0.001	NA
Language	NA	NA	NA	NA

Point estimates are pooled standardized mean differences (SMD) calculated through meta-analysis. SMDs and associated 95% confidence interval, CI, are reported each cognitive domain, along with corresponding P-values. Results are considered significant at p<0.05. Positive SMDs indicate an improvement in the treated group compared to the placebo group, whereas negative SMDs indicate the opposite effect. Estimates are reported for studies assessing estrogen therapy for surgical menopause, estrogen-progestogen therapy for spontaneous menopause, and each formulation independent of menopause type. Analyses with fewer than 10 studies are marked as not applicable (NA) for the publication bias p-values.

#### Effects of formulation and timing

**Figure 2:**
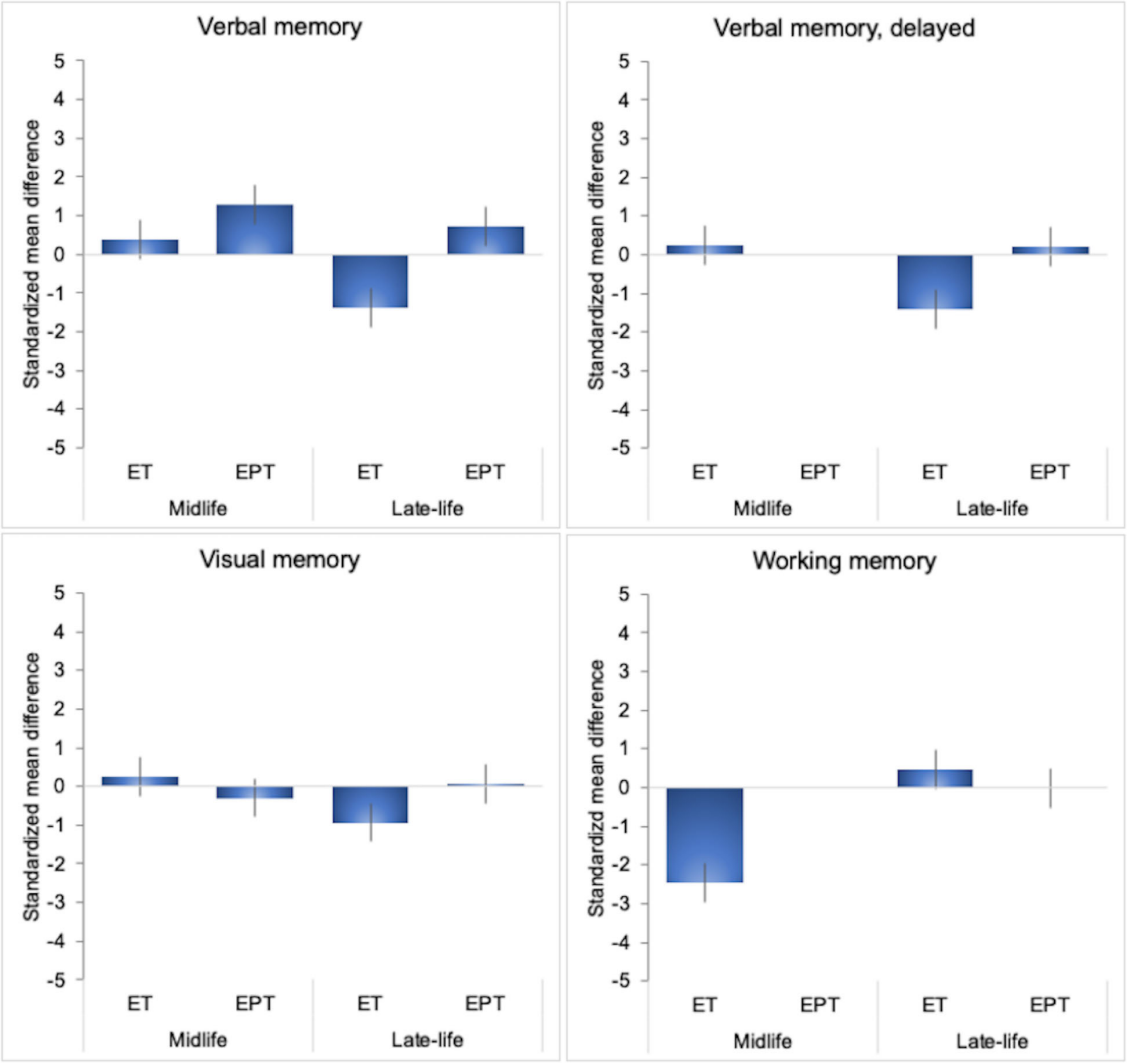
MHT effects on cognitive domains cores by initiation timing and formulation. Meta-analysis of randomized placebo-controlled trials investigating effects of systemic MHT on verbal memory, delayed verbal memory, visual memory and working memory domains. Values are standardized mean difference (SMD) and 95% confidence intervals (C.I.). ET, estrogen-only therapy; EPT, estrogen-progestogen therapy.

There were a sufficient number of studies to perform exploratory meta-analyses of verbal memory (overall and delayed recall) and visual memory domains based on type of treatment and treatment initiation. Results are summarized in **Table 6**, **Figure 2**. In midlife, we observed specific effects related to treatment type: ET was associated with mild improvements in verbal memory (SMD = 0.394, 95% CI 0.014, 0.774; *P* = 0.046), whereas EPT did not exhibit significant associations with verbal or visual memory. In late-life, effects varied: ET did not show significant effects on verbal memory or visual memory, while EPT was associated with improvements in verbal memory (*P* = 0.049) and no changes in visual memory.

**Table 6:** Effects of MHT on cognitive domains by formulation and initiation timing.

	Standardized mean difference (95% CI)	P-value	Heterogeneity p-value	Publication bias p-value
Midlife estrogen-only treatment				
Verbal memory	0.394 (0.014, 0.774)	0.046	0.429	0.705
Verbal memory, delayed	0.242 (-0.392, 0.876)	0.318	0.167	0.685
Visual memory	0.259 (-0.203, 0.721)	0.093	0.139	0.379
Midlife estrogen-progestogen treatment				
Verbal memory	1.282 (-1.973, 4.538)	0.126	0.213	NA
Verbal memory, delayed	NA	NA	NA	NA
Visual memory	-0.299 (-2.366, 1.769)	0.318	<0.001	NA
Late-life estrogen-only treatment				
Verbal memory	-1.391 (-4.034, 1.253)	0.124	0.144	0.768
Verbal memory, delayed	-1.41 (-3.371, 0.552)	0.093	0.001	NA
Visual memory	-0.935 (-3.635, 1.766)	0.348	0.001	NA
Late-life estrogen-progestogen treatment				
Verbal memory	0.71 (0.007, 1.414)	0.049	0.198	NA
Verbal memory, delayed	0.208 (-0.798, 1.214)	0.231	<0.001	NA
Visual memory	0.068 (-0.415, 0.551)	0.325	<0.001	NA

Point estimates are pooled standardized mean differences (SMD) calculated through meta-analysis. SMDs and associated 95% confidence interval, CI, are reported each cognitive domain, along with corresponding P-values. Results are considered significant at p<0.05. Positive SMDs indicate an improvement in the treated group compared to the placebo group, whereas negative SMDs indicate the opposite effect. Estimates are reported for studies assessing estrogen therapy or estrogen-progestogen therapy initiated in midlife or late-life. Analyses with fewer than 10 studies are marked as not applicable (NA) for the publication bias p-values.

### Secondary analysis: meta-analysis of individual cognitive tests

**Figure 3:**
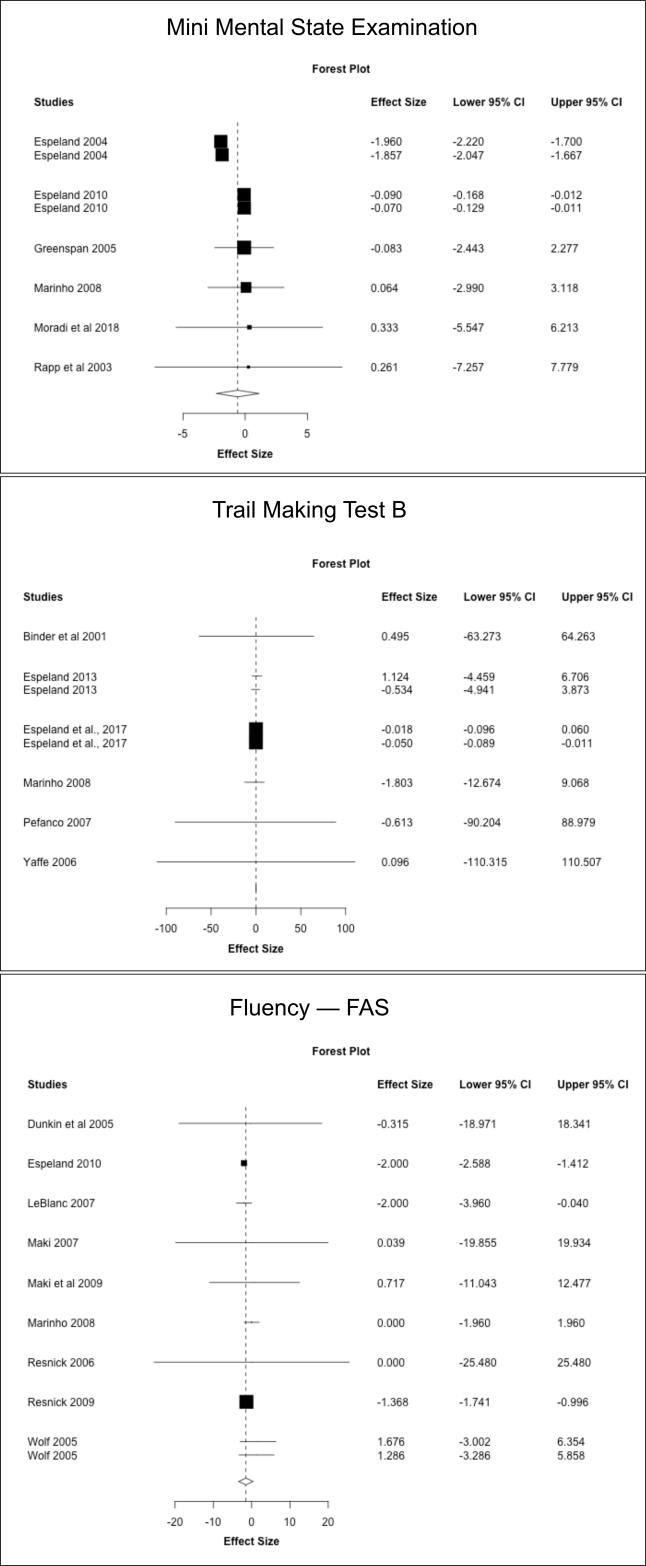
MHT effects on Mini Mental State Exam (MMSE), Trail Making Test-B, and FAS tests. Meta-analysis of randomized placebo-controlled trials investigating effects of systemic MHT on MMSE, TMT-B and FAS tests. Forest plots display individual and pooled estimates expressed as standardized mean difference (SMD) and 95% confidence intervals (C.I.). Studies are ordered by year of publication.

**Figure 4:**
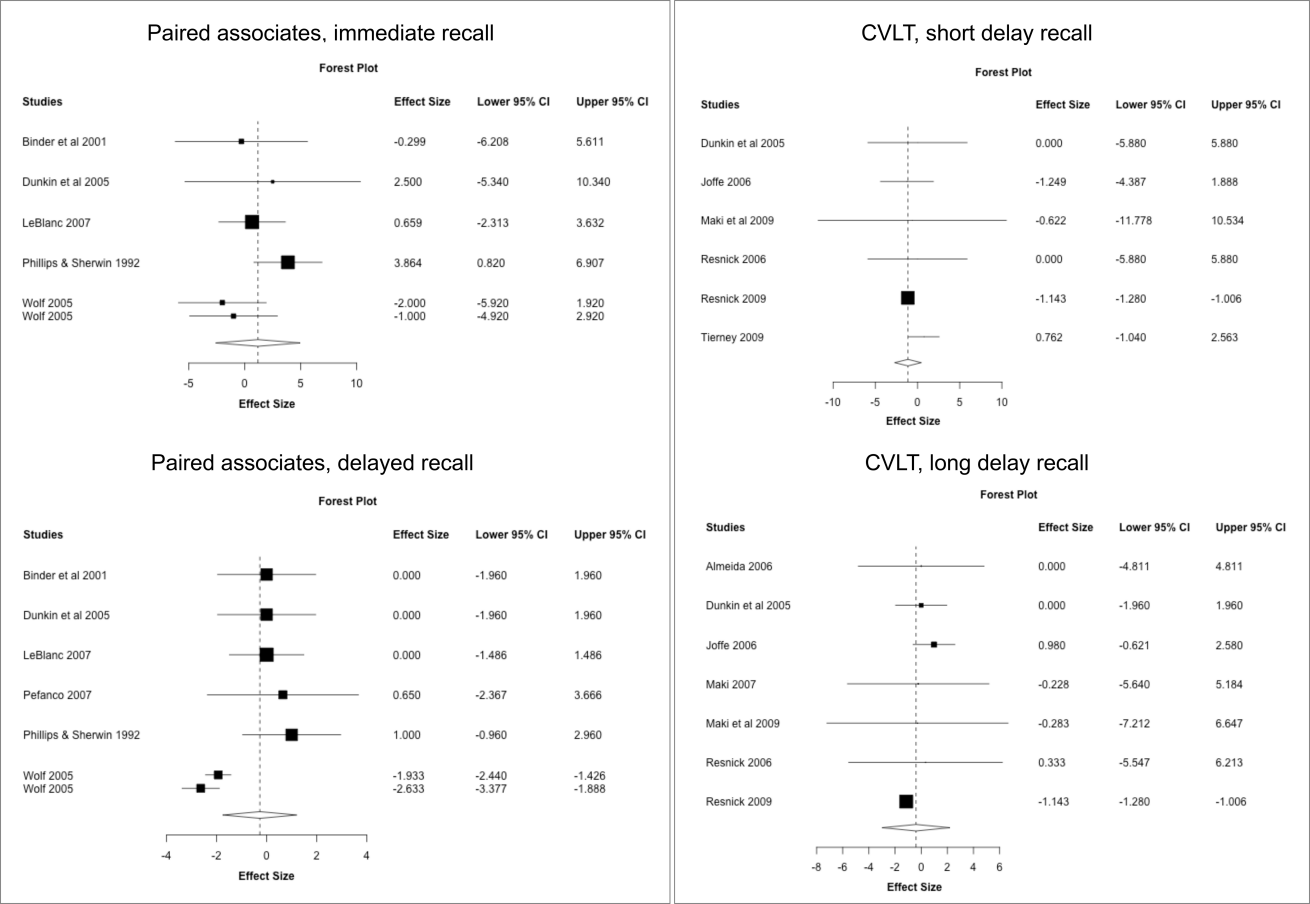
MHT effects on verbal memory tests. Meta-analysis of randomized placebo-controlled trials investigating effects of systemic MHT on paired associates immediate and delayed recall from the Wechsler memory scale, and California Verbal Learning Test (SVLT). Forest plots display individual and pooled estimates expressed as standardized mean difference (SMD) and 95% confidence intervals (C.I.). Studies are ordered by year of publication.

**Figure 5:**
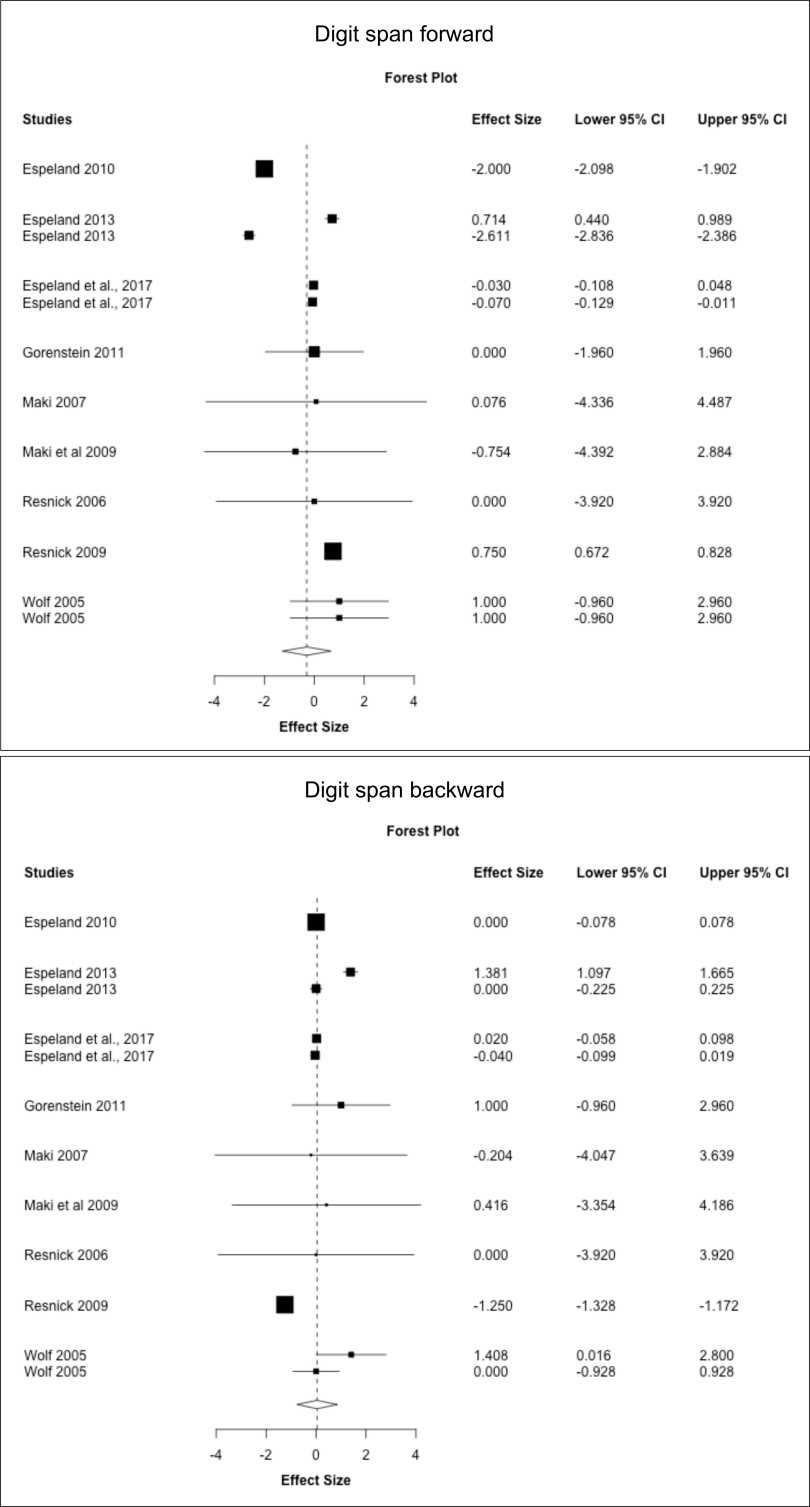
MHT effects on working memory tests. Meta-analysis of randomized placebo-controlled trials investigating effects of systemic MHT on digit span forward, backward, and total. Forest plots display individual and pooled estimates expressed as standardized mean difference (SMD) and 95% confidence intervals (C.I.). Studies are ordered by year of publication.

Next, we conducted a meta-analysis of individual cognitive tests based on data from the above RCTs. Results are summarized in **Figures 3**–**5**, **Table 7**. Pooled estimates from random-effects meta-analysis indicated a small negative effect of MHT on TMT-B compared to placebo (SMD = -0.034, 95% CI -0.041, -0.027; *P* = 0.010). As in the above analysis, excluding the two post-intervention studies had minimal impact on the results (**Table 7**).

**Table 7:** Effects of MHT on individual cognitive tests.

	Standardized mean difference (95% confidence interval)	P-value	Heterogeneity p-value	Publication bias p-value
Entire dataset				
MMSE	-0.603 (-2.313, 1.107)	0.350	0.005	NA
Paired associates, immediate	1.183 (-2.585, 4.951)	0.394	0.106	NA
Paired associates, delayed	-0.270 (-1.75, 1.210)	0.655	0.040	NA
CVLT, free recall	-1.242 (-3.075, 0.591)	0.087	<0.001	NA
CVLT, cued recall	-1.090 (-2.803, 0.623)	0.107	<0.001	NA
TMT-B	-0.034 (-0.041, -0.027)	0.010	1.343	NA
Digit span total	-0.919 (-2.763, 0.925)	0.212	<0.001	NA
Digit span forward	-0.302 (-1.28, 0.676)	0.484	<0.001	0.710
Digit span backward	0.039 (-0.77, 0.848)	0.907	<0.001	0.641
FAS	-1.507 (-3.418, 0.404)	0.067	<0.001	0.230
Excluding post-intervention studies				
MMSE	-1.393 (-6.282, 3.495)	0.258	0.194	NA
Paired associates, immediate	1.183 (-2.585, 4.951)	0.394	0.106	NA
Paired associates, delayed	-0.270 (-1.750, 1.210)	0.655	0.040	NA
CVLT, free recall	-1.131 (-2.713, 0.452)	0.070	<0.001	NA
CVLT, cued recall	-0.397 (-2.981, 2.188)	0.602	<0.001	NA
TMT-B	-0.034 (-0.041, -0.027)	0.010	1.343	NA
Digit span total	-1.200 (-3.259, 0.858)	0.153	0.035	NA
Digit span forward	0.011 (-0.936, 0.959)	0.975	<0.001	0.727
Digit span backward	0.086 (-0.903, 1.075)	0.830	<0.001	0.642
FAS	-1.324 (-2.988, 0.341)	0.064	0.001	NA

Standardized mean difference (SMD) and associated 95% confidence interval, CI, are reported each cognitive test, along with corresponding P-values. Positive SMDs indicate an improvement in the treated group compared to the placebo group, whereas negative SMDs indicate the opposite effect. Results are reported for the entire dataset and after exclusion of post-intervention studies (50, 59), at p<0.05. Analyses with fewer than 10 studies are marked as not applicable (NA) for the publication bias p-values. CVLT, California Verbal Learning Test; MMSE, Mini Mental State Examination; TMT-B, Trail Making Test B.

#### Effects of treatment duration

Pooled estimates from random effects meta-analysis indicated no changes in test scores with shorter duration of MHT treatment, and a mild worsening in TMT-B with longer treatment duration (SMD = -0.018, 95% CI -0.022, -0.014; *P* = 0.012) (**Supplementary Table 2**).

#### Effects of timing

This analysis includes estimates for studies in which treatment initiation was specified to occur in midlife or late-life (**Supplementary Table 3**). Pooled estimates from random effects meta-analysis indicated no significant differences between MHT and placebo in midlife. In contrast, MHT use in late-life was associated with small reductions in TMT-B scores (SMD = -0.050, 95% CI -0.052, -0.048; *P* = 0.002).

#### Effects of menopause type and formulation

Results are shown in **Supplementary Table 4**. ET for surgical menopause was associated with a moderate to large improvement in Digit Span forward (SMD = 0.747, 95% CI 0.603, 0.890; *P* = 0.009) but small declines in CVLT free recall (SMD = -1.142, 95% CI -1.249, -1.036; *P* = 0.005) compared to placebo. EPT for spontaneous menopause was associated with significant declines in MMSE compared to placebo (SMD = -1.853, 95% CI -2.974, -0.733; *P* = 0.030).

#### Effects of formulation and initiation timing

Results are reported in **Supplementary Table 5**. Overall, few studies offered stratification based on formulation and timing of treatment to perform a meta-analysis based on these parameters. Herein, we descriptively report results based on available data. There weren’t enough studies of midlife EPT to perform a meta-analysis. In late-life, ET was associated with improved digit span forward compared to placebo (*P* = 0.008) and had no effects on digit span backward and FAS, whereas EPT had no effects on CVLT free recall.

### Meta-regression analysis

As shown in **Supplementary Table 6**, none of the covariates had significant moderating effects on the association between MHT and cognitive performance. This suggests that none of the hypothesized confounders were the primary drivers of heterogeneity in the associations of MHT with cognition. To understand whether MHT effects on cognition were concentrated in particular cognition domains or specific tests, meta-regression models containing domain and test indicators were constructed. Model results found the executive function domain was associated with worsening in SMD as compared to the referent category of global cognition (*P* = 0.051). In analysis of individual tests, none of the tests were associated with significant changes in SMD as compared to the referent category of MMSE.

### Publication bias

No significant publication bias was observed in analysis of cognitive domains for the entire cohort or in subgroup analyses. For individual tests, only analyses of digit span forward and backward included enough studies to allow testing for publication bias. As shown in **Table 7**, no significant publication bias was observed for those tests. None of the subgroup analyses included a sufficient number of studies to evaluate presence of publication bias.

## Discussion

The present meta-analysis, examining data from 34 randomized, placebo-controlled trials encompassing 14,914 treated and 12,679 placebo participants, shows differential associations between MHT and cognitive function in some domains and tests of interest depending on formulation and timing of treatment. Overall, MHT had no significant effects on cognitive domain scores. Nonetheless, in sub-group analyses, treatment for surgical menopause, mostly using ET, improved global cognition compared to placebo. Additionally, ET initiated specifically in midlife or before age 65 was associated with improved verbal memory, while no effects were observed for ET initiated in late-life or after age 65. On the other hand, EPT was associated with MMSE declines compared to placebo, mostly with late-life treatment. However, while EPT had no significant effects in midlife, it was associated with improved verbal memory in late-life. Treatment duration >1 year was associated with worsening in visual memory as compared to shorter duration. Analysis of individual tests indicated more complex patterns of positive and negative effects of MHT with variations based on factors such as formulation and initiation timing.

### Comparison with previous meta-analyses

To date, eight meta-analyses of MHT effects on cognition have been published (35, 36, 98–101, 107, 108). Several were published before or relatively soon after the end of the WHI (35, 36, 98–100). Additionally, the majority of studies evaluated cognitive outcomes as categorical measures (e.g., improvement vs. decline vs. no change) (35, 36, 98, 99, 101), while only three studies conducted meta-analyses involving continuous measures (100, 107, 108). Among the two most recent meta-analyses, (107) examined 23 RCTs and reported a small negative effect of MHT on global cognition among postmenopausal women over 60, with no effects on memory or attention. (108) analyzed 10 RCTs, finding no significance differences in memory function between MHT and placebo, except for a negative effect on the digit span forward test among women >5 years postmenopause. No effects were observed among women ¾5 years postmenopause (108). To note, in both studies, subgroup analyses of early menopause included a maximum of 4 studies, which limits statistical power and generalizability of these findings. Possibly due to the relatively small number of studies available for analysis, neither study reported on differential effects of MHT formulation.

The present meta-analysis includes reports up to the year 2023, and takes into account known sources of heterogeneity such as formulation, timing of treatment and duration from the outset. This enabled us to integrate data across various types of studies with a large enough sample of >4 studies per subgroup. A main finding in our meta-analysis was that ET for surgical menopause had moderate positive effects on global cognition as compared to placebo, independent of initiation timing (SMD = 1.575, 95% CI 0.228, 2.921; *P* = 0.043). Additionally, when initiated specifically in midlife, ET was associated with improved verbal memory (SMD=0.394, 95% CI 0.014, 0.774; P=0.046). Cohort studies have reported an almost doubled long-term risk of dementia for women undergoing surgical menopause (11, 109–111), which was highest following bilateral oophorectomy and lower but significant with unilateral oophorectomy and partial hysterectomy (11, 35, 36, 98, 109, 110, 112). However, undergoing oophorectomy after menopause did show an increased risk of AD (113), providing supporting for the window of opportunity hypothesis. Notably, MHT initiated within 5 years post-surgery and continued for at least 10 years was also linked with less global cognitive decline (11).

### Literature Review and Interpretation Relative to the ‘Window of Opportunity’ Framework

The first RCT investigating the effects of MHT on cognitive function was the WHIMS, which evaluated CEEs and CEEs plus MPA in postmenopausal women aged 65 years or older as compared to placebo (48, 49). Contrary to the study hypotheses, dementia risk was near doubled for women in the CEE/MPA group [HR: 2.05; 95%CI: 1.21-3.48] (48), and increased by about 50% for those in the CEE only group [HR: 1.49; 95%CI: 0.83-2.66] (49). The absolute risk increase was however small, with about 12 additional cases of dementia for 1,000 women using CEE/MPA for five years, and 6 additional cases per 1,000 women using CEE for five years. Additionally, CEE therapy, alone or with MPA, was associated with mild reductions in global cognition during the first 3-4 years of follow-ups and several years later (72, 73).

The ancillary WHI Study of Cognitive Aging (WHISCA) examined whether these formulations influenced specific cognitive domains over a total of 6 years on average. A limitation of this study is that the trial addressed change in cognitive performance from an on-treatment baseline. In a sample of 2,304 postmenopausal women aged 65 and older, small changes in cognitive test scores were found (79, 84), where the CEE/MPA treated group exhibited trends towards declines in verbal learning as compared to placebo, but improved performance on figural memory testing (79), whereas no differences were observed between CEE-only and matching placebo groups (84). Additionally, two smaller trials reported no effects on cognition using oral CEEs and MPA (70) or transdermal estradiol and micronized progesterone (82).

Together, these findings suggested that CEE/MPA treatment in late-life produced an initial decrement in at least some aspects of cognitive function during the first few years of use, whereas unopposed therapy did not have positive or negative effects. Several RCTs confirmed lack of negative effects of estrogen-only therapy on memory and other cognitive outcomes in older hysterectomized women using oral estradiol, low dose transdermal estradiol (0.25 mg/day) and ultra low-dose transdermal estradiol (0.014 mg/day) (69, 76, 78, 80).

It’s important to note that RCT results from older postmenopausal women are not necessarily applicable to younger women for whom MHT is intended for. In fact, MHT appears to have generally neutral or positive effects on cognitive function in younger women, particularly improved verbal memory among surgical menopausal cases. When the effect of CEE/MPA and CEE-alone on cognition were evaluated in postmenopausal women aged 50 to 55 years, there were no differences in verbal memory, working memory, verbal fluency, and executive functions between either treated group and placebo (56). Follow-up examinations also indicated no significant long-term effects on cognitive function among younger women who had been taking hormones for up to 6 years (59). On the other hand, 65 to 79 year-old women who had been using MHT for five years or longer exhibited a decline in global cognition, executive function, and working memory, which persisted for over ten years (59). In the Cognitive Complaints in Early Menopause Trial (COGENT), with a sample size of 180 women aged 45–55 years of age, there were no significant effects of CEE/MPA on memory performance after 4 months of treatment, although trends were noted toward declines in short and long-delay verbal memory compared to placebo (81). Similar results were found in a trial of women with moderate-to-severe hot flashes study who were randomized to 1 year treatment with CEE/MPA, black cohosh, or red clover vs. placebo (67). The CEE/MPA group showed some improvement in verbal fluency test but worsening in some memory tests compared to placebo (67).

Additional studies provided similar results with different MHT formulations. Among women aged 42–58 years, who were within 3 years of their final menstrual period, 4-year treatment with oral CEE (0.45 mg/day) or transdermal estradiol (50 microg weekly) with or without cyclic micronized progesterone (200 mg for 12 days each month) showed no changes compared to placebo (57). Likewise, after an average of 57 months of treatment, oral estradiol with or without micronized progesterone administered to postmenopausal women divided into early (mean age of 56 years) and late postmenopausal (mean age of 65 years), resulted in no effects on verbal memory, executive function, or global cognition compared to placebo (58).

Several smaller studies have also been conducted, showing either neutral or protective effects of MHT in younger surgically menopausal women (114–116, 60, 65). Studies examining the cognitive effects of opposed therapy in younger women with a uterus are scarcer and report contrasting results. For example, EPT was associated with improvement in some memory tests but worsening on some executive function tests in one study (86) and with neutral effects in another study (66). One RCT that directly compared estradiol valerate to estradiol valerate plus dienogest and placebo showed a benefit of opposed therapy on verbal memory compared with both placebo and estradiol (71). This suggests that different forms of progestogen may have different effects on cognition, with dienogest exhibiting more positive or neutral effects than MPA.

The notion that estrogen therapy administered to midlife women may be beneficial to some aspects of cognition was supported by observational evidence of positive effects of midlife MHT use on cognition and AD risk (35–46). For instance, in the prospective Cache County study, use of hormone therapy during the menopausal window was protective against AD, whereas use after age 61 was associated with 2.1-fold increased risk (41). Additionally, women who initiated MHT within five years of menopause exhibited better cognitive performance than those who initiated treatment alter on (117). Similar results were found in the Kaiser Permanente study, where women who used MHT in midlife had a 26% reduced risk of dementia compared to never-users, while those who used MHT in late-life experienced a 48% higher risk of dementia (43). While observational studies are subject to several limitations, the majority show that estrogen may help maintain some aspects of cognition (36, 118–122), particularly verbal memory (52, 98, 123), when initiated early in the course of menopause.

### Limitations and implications for future research

A limitation of this meta-analysis was the relatively small number of studies included in each subgroup analysis. Out of the 52 RCTs initially identified, only 34 provided continuous estimates suitable for meta-analysis. Additionally, lack of standardization and use of several different cognitive tests across studies limited data integration. To address this issue, we focused our primary analyses on cognitive domains rather than individual tests, thus maximizing available data. It is important to clarify that we use the term ‘domain’ to refer to groupings of specific cognitive tests assessing the same cognitive function. We are thus not creating novel cognitive constructs but rather examining standardized data from different cognitive tests within a unified category, so as to streamline analysis by considering related tests collectively. Our aim was to provide a broader perspective on cognitive function, which could have mitigated the impact of specific test-level variations, while enhancing statistical power. This approach, which was used in some previous meta-analyses (36, 98–101), likely allowed us to capture a more comprehensive view of cognitive performance, potentially smoothing out the fluctuations seen at the individual test level. However, it may have conservatively reduced our power to detect significant effects of MHT on cognition. As different studies categorize certain tests differently, we based our grouping on generally accepted criteria as well as insights from prior studies (36, 98–101). Nonetheless, more research is needed to confirm the validity of these groupings within the context of MHT and cognition.

For completeness, meta-analysis of individual cognitive tests was provided as a secondary analysis. This analysis was limited by the relatively smaller number of studies reporting on the same cognitive tests. While most of the results did not reach statistical significance, a few negative and positive effects were observed. MHT was associated with a worsening in TMT-B scores relative to placebo, which was associated with studies of late-life use and longer duration of use. EPT was associated with MMSE declines compared to placebo, while ET was associated with improvements in Digit Span forward, particularly among studies of late-life treatment. However, there were also small declines in CVLT free recall among ET users when compared to placebo. Caution is needed when interpreting these results, as certain large-scale ancillary studies within the WHI (59, 84) were heavily weighted in these sub-analyses and had a substantial impact on the pooled estimates. Overall, these findings underscore the variable effects of MHT on different cognitive tests, with outcomes influenced by factors such as the type of therapy, timing of initiation, and treatment duration.

Another limitation relates to sample size disparities within the subgroups. The sample size for studies involving early menopause was smaller compared to those focusing on late-life interventions. This discrepancy may have reduced our power to detect significant effects within the early menopause subgroup. Additionally, the heterogeneity detected in the meta-analyses suggests the need for a more detailed investigation into methodological variations across studies. We addressed heterogeneity by performing both subgroup analyses and multi-level meta-regressions assessing the impact of type of menopause, MHT formulation, initiation timing and treatment duration. While these analyses revealed interesting patterns of MHT effects, the persistence of significant heterogeneity evident in meta-regression analyses accounting for these factors suggests the influence of additional unaccounted factors. For instance, as with previous meta-analyses (107, 108), the insufficient number of included studies hindered further examinations of different types of estrogen (estradiol vs. CEE) or progestogen (synthetic progestins vs. micronized progesterone), and route of administration (oral, transdermal, or intramuscular), as well as between continuous vs. cyclic use of progestogen. These factors could yield valuable insights into the nuances of MHT effects on cognition. Additionally, variables such as education and socioeconomical status, which also can increase heterogeneity, were accounted for in many the studies, and we used the fully adjusted estimates in our meta-analysis. However, possible risks for menopause-associated cognitive decline such as smoking status, comorbidities like diabetes or thyroid disease, and lifestyle factors were not consistently screened for or accounted for in the included studies and could have contributed to the observed heterogeneity. Overall, presence of residual heterogeneity is an indication of limitations within the existing literature. While this does not undermine the validity of the analysis, it does emphasize the need for caution in interpreting the findings.

Finally, when conducting multiple meta-analyses, there is a potential for a multiple comparisons issue. We did not apply a correction for multiple comparisons because our study design *a priori* included the main meta-analysis followed by sensitivity analyses encompassing distinct questions, each focusing on different aspects of the relationship between MHT and cognition, such as timing, formulation, and type of menopause. Therefore, we frame our analysis as exploratory and hypothesis-generating, rather than confirmatory, with the primary aim of providing an overview of the available evidence. Consequently, our findings do not warrant clinical application but rather support research interest in MHT for cognitive support and underscore the need for more comprehensive research in this area.

While clinical trials utilizing standardized cognitive tests as primary endpoints offer valuable insights, some limitations should also be considered. These limitations encompass statistical, methodological, and conceptual aspects. One notable limitation is the small sample sizes (<1,000 participants) observed in 28 out of the 34 studies. This may constrain statistical power, thus hindering the ability to identify statistically significant effects, particularly when exploring cognitive tests and domains that exhibit subtle changes. Cognitive performance can also be influenced by practice effects, particularly when trials are relatively brief. Participants’ familiarity with cognitive tests may lead to improvements in scores over time, irrespective of the treatment under investigation, which can mask or confound the true cognitive impact of MHT.

The variation in cognitive test selection and sensitivity across different studies is another limitation. Some trials focused exclusively on one or few specific cognitive tests or specific components of a test. This selectivity in test choice may not fully capture the multifaceted nature of cognitive function, potentially overlooking effects that may be apparent in a broader cognitive assessment. Further, certain cognitive tests employed may not be inherently sensitive to hormone levels. For instance, several studies used the MMSE or similar global cognition test as their primary (and sometimes only) endpoint, although there is no clear evidence that these tests would be sensitive to estrogen changes. The variability in cognitive tests employed across studies is another possible source of heterogeneity. These discrepancies encompass factors such as variations in test length, difficulty, word relatedness, the use of early acquired or frequently used language, repeated administration of the same test or parallel versions (with concerns about reliability and comparability), as well as potential differences among testers in multi-setting studies. Finally, it remains to be established whether the observed changes in cognition were primarily due to the effects of MHT or the remission of menopausal symptoms following treatment.

The study of MHT’s impact on cognition faces some basic challenges. Firstly, existing evidence suggests that estrogen therapy primarily maintains cognitive function rather than enhancing it. For instance, in surgical menopause, estrogen suppression has been linked to memory deficits, with subsequent treatment restoring performance to baseline levels (60, 115, 116). This creates a statistical hurdle, as it essentially requires the placebo group to deteriorate to a sufficient degree for the treatment group’s benefits to be statistically detectable. If the placebo group’s cognitive performance remains relatively stable or does not decline significantly, it is challenging to demonstrate improvement in the treatment group, even if estrogen therapy is indeed preserving cognitive function effectively. To this point, another important consideration arises from evidence that cognitive performance doesn’t appear to be impaired during menopause (124, 125). In statistical terms, if there is no pre-existing cognitive deficit, it may be impractical to expect MHT to improve cognitive function. Additionally, ‘ceiling effects’ may come into play when cognitive performance is already near the upper limits of measurement sensitivity, making it challenging to detect further improvements, even if they exist. This presents a conceptual challenge, as MHT interventions aim to enhance or maintain cognitive abilities, which may already be functioning at a satisfactory level. Further, although cognitive complaints are generally more frequent in the perimenopausal stage early postmenopausal than in the late postmenopausal stages (124–126), no clinical trials have focused specifically on perimenopausal women. For these reasons, it may be challenging to detect a signal for estrogen benefits on cognition in postmenopausal women. Overall, more studies are needed that carefully consider the baseline cognitive status of participants, the sensitivity of selected cognitive endpoints, and the appropriateness of cognitive improvement as an achievable outcome.

Studies that incorporate biological markers of AD are also warranted to overcome some limitations of existing literature. First off, increasing research identifies increased biomarker indicators of AD risk among perimenopausal and postmenopausal women compared to premenopausal controls or age-controlled men, including higher amyloid-beta (Aβ) deposition (11–16) and tau pathology (127), glucose hypometabolism (12–15, 128) and lower gray matter volume in some AD-vulnerable regions (12–15, 128–131) Additionally, some studies report altered mitochondrial energy production (132, 133) and more white matter hyperintensities (134) in postmenopausal women.

While neuroimaging studies of MHT are scarce, several indicate a possible beneficial role of estrogen therapy on biomarkers such as glucose metabolism (14, 135–138), cerebral blood flow (139, 140), Aβ deposition (14, 141) and tau pathology (142). Notably, a recent prospective study reported, over a 6-month period, a smaller reduction in Aβ_42_/p-tau_231_ in MHT users compared to non-users (143). Medical imaging examining presence of cerebrovascular and neurodegenerative insults prior to treatment initiation can further guide both research efforts in identifying the optimal treatment timeline, providing important information for individualized treatment.

Finally, the type of progestogen used in MHT formulations is also of interest. Various progestogens have been utilized in MHT preparations, including several synthetic progestins such as MPA, norethindrone, norethisterone, drospirenone, and dienogest. There is laboratory evidence that certain progestins, particularly MPA, may antagonize the effects of estrogen on brain regions involved in memory function, such as the hippocampus (144, 145). Additionally, MPA users exhibit a higher risk of breast cancer and venous thromboembolism as compared to users of dydrogesterone (146). This suggests that the progestin type may modulate the impact of opposed therapy on some cognitive functions. Oral MPA was the most common progestogen used in the studies included in this meta-analysis, which may have contributed to the declines in global cognition observed in late-life.

In the end, our findings highlight the need for further research in this area, preferably by means of clinical trials using cognitive tests and AD biomarkers as endpoints. Moreover, the inclusion of a larger number of studies, especially those reporting continuous estimates, will enhance the statistical power and generalizability of findings. Stratified analyses based on estrogen and progestogen types initiated in midlife, and with different treatment durations, are warranted. For clinical practice, achieving precision hormone therapy will likely require *a priori* identification of women appropriate for MHT and the type and dose of MHT appropriate for their individual characteristics and health risks (146).

### Conclusions

This meta-analysis suggests time-dependent effects of MHT on certain aspects of cognition, with variations based on formulation and timing of initiation, underscoring the need for further research with larger samples and more homogeneous study designs. Continued research is needed to further refine our understanding of these effects, enabling more informed clinical decisions for menopausal women seeking cognitive benefits from MHT.

## Data availability statement

The original contributions presented in the study are included in the article/**Supplementary Material**. Further inquiries can be directed to the corresponding author.

## Author contributions

CA: Data curation, Formal analysis, Investigation, Methodology, Visualization, Writing – original draft. MN: Data curation, Investigation, Writing – review & editing. SJ: Data curation, Investigation, Writing – review & editing. CC: Data curation, Writing – review & editing. CZ: Writing – review & editing. CB: Writing – review & editing. FF: Writing – review & editing. TA: Writing – review & editing. MB: Writing – review & editing. SP: Writing – review & editing. PC: Writing – review & editing. SW: Writing – review & editing, Supervision. MF: Writing – review & editing. RB: Writing – review & editing. LM: Conceptualization, Funding acquisition, Project administration, Resources, Supervision, Writing – original draft, Writing – review & editing.

## References

[1] Alzheimer’s Association . 2022 Alzheimer’s disease facts and figures. Alzheimers Dement. (2022) 18:700–89. doi: 10.1002/alz.12638 35289055

[2] Farrer LA Cupples LA Haines JL Hyman B Kukull WA Mayeux R . Effects of age, sex, and ethnicity on the association between apolipoprotein E genotype and Alzheimer disease. A meta-analysis. APOE and Alzheimer Disease Meta Analysis Consortium. Jama. (1997) 278:1349–56. doi: 10.1001/jama.278.16.1349 9343467

[3] Altmann A Tian L Henderson VW Greicius MD Alzheimer’s Disease Neuroimaging Initiative I . Sex modifies the APOE-related risk of developing Alzheimer disease. Ann Neurol. (2014) 75:563–73. doi: 10.1002/ana.24135 PMC4117990 24623176

[4] Gao S Hendrie HC Hall KS Hui S . The relationships between age, sex, and the incidence of dementia and Alzheimer disease: a meta-analysis. Arch Gen Psychiatry. (1998) 55:809–15. doi: 10.1001/archpsyc.55.9.809 9736007

[5] Andersen K Launer LJ Dewey ME Letenneur L Ott A Copeland JR . Gender differences in the incidence of AD and vascular dementia: The EURODEM Studies. EURODEM Incidence Research Group. Neurology. (1999) 53:1992–7. doi: 10.1212/WNL.53.9.1992 10599770

[6] Fratiglioni L Launer LJ Andersen K Breteler MM Copeland JR Dartigues JF . Incidence of dementia and major subtypes in Europe: A collaborative study of population-based cohorts. Neurologic Diseases in the Elderly Research Group. Neurology. (2000) 54:S10–15. 10854355

[7] Lobo A Launer LJ Fratiglioni L Andersen K Di Carlo A Breteler MM . Prevalence of dementia and major subtypes in Europe: A collaborative study of population-based cohorts. Neurologic Diseases in the Elderly Research Group. Neurology. (2000) 54:S4–9. 10854354

[8] Ferretti MT Iulita MF Cavedo E Chiesa PA Schumacher Dimech A Santuccione Chadha A . Sex differences in Alzheimer disease - the gateway to precision medicine. Nat Rev Neurol. (2018) 14:457–69. doi: 10.1038/s41582-018-0032-9 29985474

[9] Rahman A Jackson H Hristov H Isaacson RS Saif N Shetty T . Sex and gender driven modifiers of alzheimer’s: the role for estrogenic control across age, race, medical, and lifestyle risks. Front Aging Neurosci. (2019) 11:315. doi: 10.3389/fnagi.2019.00315 31803046 PMC6872493

[10] Sperling RA Karlawish J Johnson KA . Preclinical Alzheimer disease-the challenges ahead. Nat Rev Neurol. (2013) 9:54–8. doi: 10.1038/nrneurol.2012.241 PMC3643203 23183885

[11] Bove R Secor E Chibnik LB Barnes LL Schneider JA Bennett DA . Age at surgical menopause influences cognitive decline and Alzheimer pathology in older women. Neurology. (2014) 82:222–9. doi: 10.1212/WNL.0000000000000033 PMC3902759 24336141

[12] Mosconi L Berti V Quinn C Mchugh P Petrongolo G Varsavsky I . Sex differences in Alzheimer risk: Brain imaging of endocrine vs chronologic aging. Neurology. (2017) 89:1382–90. doi: 10.1212/WNL.0000000000004425 PMC5652968 28855400

[13] Mosconi L Rahman A Diaz I Wu X Scheyer O Hristov HW . Increased Alzheimer’s risk during the menopause transition: A 3-year longitudinal brain imaging study. PloS One. (2018) 13:e0207885. doi: 10.1371/journal.pone.0207885 30540774 PMC6291073

[14] Rahman A Schelbaum E Hoffman K Diaz I Hristov H Andrews R . Sex-driven modifiers of Alzheimer risk. Neurology. (2020) 95:e166. doi: 10.1212/WNL.0000000000009781 32580974 PMC7455325

[15] Mosconi L Berti V Dyke J Schelbaum E Jett S Loughlin L . Menopause impacts human brain structure, connectivity, energy metabolism, and amyloid-beta deposition. Sci Rep. (2021) 11:10867. doi: 10.1038/s41598-021-90084-y 34108509 PMC8190071

[16] Coughlan GT Betthauser TJ Boyle R Koscik RL Klinger HM Chibnik LB . Association of Age at Menopause and Hormone Therapy Use With Tau and beta-Amyloid Positron Emission Tomography. JAMA Neurol. (2023) 80:462–73. doi: 10.1001/jamaneurol.2023.0455 PMC10071399 37010830

[17] Goodman Y Bruce AJ Cheng B Mattson MP . Estrogens attenuate and corticosterone exacerbates excitotoxicity, oxidative injury, and amyloid beta-peptide toxicity in hippocampal neurons. J Neurochem. (1996) 66:1836–44. doi: 10.1046/j.1471-4159.1996.66051836.x 8780008

[18] Mcewen BS Alves SE Bulloch K Weiland NG . Ovarian steroids and the brain: implications for cognition and aging. Neurology. (1997) 48:S8–15. doi: 10.1212/WNL.48.5_Suppl_7.8S 9153161

[19] Brinton RD Yao J Yin F Mack WJ Cadenas E . Perimenopause as a neurological transition state. Nat Rev Endocrinol. (2015) 11:393–405. doi: 10.1038/nrendo.2015.82 26007613 PMC9934205

[20] Hara Y Waters EM Mcewen BS Morrison JH . Estrogen effects on cognitive and synaptic health over the lifecourse. Physiol Rev. (2015) 95:785–807. doi: 10.1152/physrev.00036.2014 26109339 PMC4491541

[21] Lai YJ Yu D Zhang JH Chen GJ . Cooperation of genomic and rapid nongenomic actions of estrogens in synaptic plasticity. Mol Neurobiol. (2017) 54:4113–26. doi: 10.1007/s12035-016-9979-y PMC5509832 27324789

[22] Brinton RD . The healthy cell bias of estrogen action: mitochondrial bioenergetics and neurological implications. Trends Neurosci. (2008) 31:529–37. doi: 10.1016/j.tins.2008.07.003 PMC10124615 18774188

[23] Arevalo M-A Azcoitia I Garcia-Segura LM . The neuroprotective actions of oestradiol and oestrogen receptors. Nat Rev Neurosci. (2015) 16:17–29. doi: 10.1038/nrn3856 25423896

[24] Jett S Malviya N Schelbaum E Jang G Jahan E Clancy K . Endogenous and exogenous estrogen exposures: how women’s reproductive health can drive brain aging and inform alzheimer’s prevention. Front Aging Neurosci. (2022) 14:831807. doi: 10.3389/fnagi.2022.831807 35356299 PMC8959926

[25] Jett S Schelbaum E Jang G Boneu Yepez C Dyke JP Pahlajani S . Ovarian steroid hormones: A long overlooked but critical contributor to brain aging and Alzheimer’s disease. Front Aging Neurosci. (2022) 14:948219. doi: 10.3389/fnagi.2022.948219 35928995 PMC9344010

[26] Morrison JH Brinton RD Schmidt PJ Gore AC . Estrogen, menopause, and the aging brain: how basic neuroscience can inform hormone therapy in women. J Neurosci. (2006) 26:10332–48. doi: 10.1523/JNEUROSCI.3369-06.2006 PMC6674699 17035515

[27] Livingston G Huntley J Sommerlad A Ames D Ballard C Banerjee S . Dementia prevention, intervention, and care: 2020 report of the Lancet Commission. Lancet. (2020) 396:413–46. doi: 10.1016/S0140-6736(20)30367-6 PMC7392084 32738937

[28] Davis SR Lambrinoudaki I Lumsden M Mishra GD Pal L Rees M . Menopause. Nat Rev Dis Primers. (2015) 1:15004. doi: 10.1038/nrdp.2015.4 27188659

[29] Monteleone P Mascagni G Giannini A Genazzani AR Simoncini T . Symptoms of menopause — global prevalence, physiology and implications. Nat Rev Endocrinol. (2018) 14:199–215. doi: 10.1038/nrendo.2017.180 29393299

[30] El Khoudary SR Greendale G Crawford SL Avis NE Brooks MM Thurston RC . The menopause transition and women’s health at midlife: a progress report from the Study of Women’s Health Across the Nation (SWAN). Menopause. (2019) 26:1213–27. doi: 10.1097/GME.0000000000001424 PMC6784846 31568098

[31] Linn RT Wolf PA Bachman DL Knoefel JE Cobb JL Belanger AJ . The ‘preclinical phase’ of probable Alzheimer’s disease. A 13-year prospective study of the Framingham cohort. Arch Neurol. (1995) 52:485–90. doi: 10.1001/archneur.1995.00540290075020 7733843

[32] Petersen RC . Mild cognitive impairment as a diagnostic entity. J Intern Med. (2004) 256:183–94. doi: 10.1111/j.1365-2796.2004.01388.x 15324362

[33] Tierney MC Yao C Kiss A Mcdowell I . Neuropsychological tests accurately predict incident Alzheimer disease after 5 and 10 years. Neurology. (2005) 64:1853–9. doi: 10.1212/01.WNL.0000163773.21794.0B 15955933

[34] Jack CR Jr. Knopman DS Weigand SD Wiste HJ Vemuri P Lowe V . An operational approach to National Institute on Aging-Alzheimer’s Association criteria for preclinical Alzheimer disease. Ann Neurol. (2012) 71:765–75. doi: 10.1002/ana.22628 PMC3586223 22488240

[35] Yaffe K Sawaya G Lieberburg I Grady D . Estrogen therapy in postmenopausal women: effects on cognitive function and dementia. JAMA. (1998) 279:688–95. doi: 10.1001/jama.279.9.688 9496988

[36] Hogervorst E Williams J Budge M Riedel W Jolles J . The nature of the effect of female gonadal hormone replacement therapy on cognitive function in post-menopausal women: a meta-analysis. Neuroscience. (2000) 101:485–512. doi: 10.1016/S0306-4522(00)00410-3 11113299

[37] Paganini-Hill A Henderson VW . Estrogen replacement therapy and risk of Alzheimer disease. Arch Intern Med. (1996) 156:2213–7. doi: 10.1001/archinte.156.19.2213 8885820

[38] Tang MX Jacobs D Stern Y Marder K Schofield P Gurland B . Effect of oestrogen during menopause on risk and age at onset of Alzheimer’s disease. Lancet. (1996) 348:429–32. doi: 10.1016/S0140-6736(96)03356-9 8709781

[39] Kawas C Resnick S Morrison A Brookmeyer R Corrada M Zonderman A . A prospective study of estrogen replacement therapy and the risk of developing Alzheimer’s disease: the Baltimore Longitudinal Study of Aging. Neurology. (1997) 48:1517–21. doi: 10.1212/WNL.48.6.1517 9191758

[40] Slooter AJ Bronzova J Witteman JC Van Broeckhoven C Hofman A Van Duijn CM . Estrogen use and early onset Alzheimer’s disease: a population-based study. J Neurology Neurosurg Psychiatry. (1999) 67:779–81. doi: 10.1136/jnnp.67.6.779 PMC1736655 10567497

[41] Zandi PP Carlson MC Plassman BL Welsh-Bohmer KA Mayer LS Steffens DC . Hormone replacement therapy and incidence of Alzheimer disease in older women: the Cache County Study. Jama. (2002) 288:2123–9. doi: 10.1001/jama.288.17.2123 12413371

[42] Henderson VW Benke K Green R Cupples L Farrer L . Postmenopausal hormone therapy and Alzheimer’s disease risk: interaction with age. J Neurology Neurosurg Psychiatry. (2005) 76:103–5. doi: 10.1136/jnnp.2003.024927 PMC1739309 15608005

[43] Whitmer RA Quesenberry CP Zhou J Yaffe K . Timing of hormone therapy and dementia: the critical window theory revisited. Ann Neurol. (2011) 69:163–9. doi: 10.1002/ana.22239 PMC3058824 21280086

[44] Shao H Breitner JC Whitmer RA Wang J Hayden K Wengreen H . Hormone therapy and Alzheimer disease dementia: new findings from the Cache County Study. Neurology. (2012) 79:1846–52. 10.1212/WNL.0b013e318271f823 PMC3525314 23100399

[45] Yoo J Shin D Han K Kim D Won HS Lee J . Female reproductive factors and the risk of dementia: a nationwide cohort study. Eur J Neurol. (2020) 27:1448–58. doi: 10.1111/ene.14315 32396982

[46] Kim YJ Soto M Branigan GL Rodgers K Brinton RD . Association between menopausal hormone therapy and risk of neurodegenerative diseases: Implications for precision hormone therapy. Alzheimers Dement (N Y). (2021) 7:e12174. doi: 10.1002/trc2.12174 34027024 PMC8118114

[47] Nerattini M Jett S Andy C Carlton C Zarate C Boneu C . Systematic review and meta-analysis of the effects of menopause hormone therapy on risk of Alzheimer’s disease and dementia. Front Aging Neurosci. (2023) 15. doi: 10.3389/fnagi.2023.1260427 PMC10625913 37937120

[48] Shumaker SA Legault C Rapp SR Thal L Wallace RB Ockene JK . Estrogen plus progestin and the incidence of dementia and mild cognitive impairment in postmenopausal women: the Women’s Health Initiative Memory Study: a randomized controlled trial. JAMA. (2003) 289:2651–62. doi: 10.1001/jama.289.20.2651 12771112

[49] Shumaker SA Legault C Kuller L Rapp SR Thal L Lane DS . Conjugated equine estrogens and incidence of probable dementia and mild cognitive impairment in postmenopausal women: Women’s Health Initiative Memory Study. JAMA. (2004) 291:2947–58. doi: 10.1001/jama.291.24.2947 15213206

[50] Espeland Ma BR Hogan Pa Rapp Sr Coker Lh Legault C Granek I . Long term effects of conjugated equine estrogens therapies on domain-specific cognitive function: results from the women’s health initiative study of cognitive aging (WHISCA) extension. J Am Geriatr Soc. (2010) 28:1263–71. 10.1111/j.1532-5415.2010.02953.x PMC2917208 20649689

[51] Maki PM . The timing of estrogen therapy after ovariectomy–implications for neurocognitive function. Nat Clin Pract Endocrinol Metab. (2008) 4:494+. doi: 10.1038/ncpendmet0901 18648334

[52] Maki PM Sundermann E . Hormone therapy and cognitive function. Hum Reprod Update. (2009) 15:667–81. doi: 10.1093/humupd/dmp022 PMC2759330 19468050

[53] Henderson VW Rocca WA . Estrogens and Alzheimer disease risk: Is there a window of opportunity. American Academy of Neurology (AAN) (2012). doi: 10.1212/WNL.0b013e318271f88f 23100400

[54] Rossouw JE Manson JE Kaunitz AM Anderson GL . Lessons learned from the Women’s Health Initiative trials of menopausal hormone therapy. Obstet Gynecol. (2013) 121:172–6. doi: 10.1097/AOG.0b013e31827a08c8 PMC3547645 23262943

[55] Manson JE Bassuk SS Kaunitz AM Pinkerton JV . The Women’s Health Initiative trials of menopausal hormone therapy: lessons learned. Menopause. (2020) 27:918–28. doi: 10.1097/GME.0000000000001553 32345788

[56] Espeland MA Shumaker SA Leng I Manson JE Brown CM Leblanc ES . Long-term effects on cognitive function of postmenopausal hormone therapy prescribed to women aged 50 to 55 years. JAMA Intern Med. (2013) 173:1429–36. doi: 10.1001/jamainternmed.2013.7727 PMC3844547 23797469

[57] Gleason CE Dowling NM Wharton W Manson JE Miller VM Atwood CS . Effects of hormone therapy on cognition and mood in recently postmenopausal women: findings from the randomized, controlled KEEPS-cognitive and affective study. PloS Med. (2015) 12:e1001833; discussion e1001833. doi: 10.1371/journal.pmed.1001833 26035291 PMC4452757

[58] Henderson VW St John JA Hodis HN Mccleary CA Stanczyk FZ Shoupe D . Cognitive effects of estradiol after menopause: A randomized trial of the timing hypothesis. Neurology. (2016) 87:699–708. doi: 10.1212/WNL.0000000000002980 27421538 PMC4999165

[59] Espeland MA Rapp SR Manson JE Goveas JS Shumaker SA Hayden KM . Long-term effects on cognitive trajectories of postmenopausal hormone therapy in two age groups. J Gerontol A Biol Sci Med Sci. (2017) 72:838–45. doi: 10.1093/gerona/glw156 PMC6075542 27506836

[60] Phillips SM Sherwin BB . Effects of estrogen on memory function in surgically menopausal women. Psychoneuroendocrinology. (1992) 17:485–95. doi: 10.1016/0306-4530(92)90007-T 1484915

[61] Duka T Tasker R Mcgowan JF . The effects of 3-week estrogen hormone replacement on cognition in elderly healthy females. Psychopharmacol (Berl). (2000) 149:129–39. doi: 10.1007/s002139900324 10805607

[62] Shaywitz SE Naftolin F Zelterman D Marchione KE Holahan JM Palter SF . Better oral reading and short-term memory in midlife, postmenopausal women taking estrogen. Menopause. (2003) 10:420–6. doi: 10.1097/01.GME.0000060241.02837.29 14501603

[63] Joffe H Hall JE Gruber S Sarmiento IA Cohen LS Yurgelun-Todd D . Estrogen therapy selectively enhances prefrontal cognitive processes: a randomized, double-blind, placebo-controlled study with functional magnetic resonance imaging in perimenopausal and recently postmenopausal women. Menopause. (2006) 13:411–22. doi: 10.1097/01.gme.0000189618.48774.7b 16735938

[64] Leblanc ES Neiss MB Carello PE Samuels MH Janowsky JS . Hot flashes and estrogen therapy do not influence cognition in early menopausal women. Menopause. (2007) 14:191–202. doi: 10.1097/01.gme.0000230347.28616.1c 17194963

[65] Gorenstein C Rennó J Jr. Vieira Filho AH Gianfaldoni A Gonçalves MA Halbe HW . Estrogen replacement therapy and cognitive functions in healthy postmenopausal women: a randomized trial. Arch Womens Ment Health. (2011) 14:367–73. doi: 10.1007/s00737-011-0230-6 21732218

[66] Moradi F Jahanian Sadatmahalleh S Ziaei S . The effect of hormone replacement therapy on cognitive function in postmenopausal women: An RCT. Int J Reprod BioMed. (2019) 16(12):ijrm.v16i12.3682. doi: 10.18502/ijrm.v16i12.3682 31417982 PMC6600282

[67] Maki PM Rubin LH Fornelli D Drogos L Banuvar S Shulman LP . Effects of botanicals and combined hormone therapy on cognition in postmenopausal women. Menopause. (2009) 16:1167–77. doi: 10.1097/gme.0b013e3181ace484 PMC2783198 19590458

[68] Page MJ Mckenzie JE Bossuyt PM Boutron I Hoffmann TC Mulrow CD . The PRISMA 2020 statement: an updated guideline for reporting systematic reviews. BMJ. (2021) 372:n71. doi: 10.1136/bmj.n71 33782057 PMC8005924

[69] Wolf OT Kudielka BM Hellhammer DH Torber S Mcewen BS Kirschbaum C . Two weeks of transdermal estradiol treatment in postmenopausal elderly women and its effect on memory and mood: verbal memory changes are associated with the treatment induced estradiol levels. Psychoneuroendocrinology. (1999) 24:727–41. doi: 10.1016/S0306-4530(99)00025-6 10451908

[70] Binder EF Schechtman KB Birge SJ Williams DB Kohrt WM . Effects of hormone replacement therapy on cognitive performance in elderly women. Maturitas. (2001) 38:137–46. doi: 10.1016/S0378-5122(00)00214-0 11306202

[71] Linzmayer L Semlitsch HV Saletu B Bock G Saletu-Zyhlarz G Zoghlami A . Double-blind, placebo-controlled psychometric studies on the effects of a combined estrogen-progestin regimen versus estrogen alone on performance, mood and personality of menopausal syndrome patients. Arzneimittelforschung. (2001) 51:238–45. doi: 10.1055/s-0031-1300030 11304940

[72] Rapp SR Espeland MA Shumaker SA Henderson VW Brunner RL Manson JE . Effect of estrogen plus progestin on global cognitive function in postmenopausal women: the Women’s Health Initiative Memory Study: a randomized controlled trial. JAMA. (2003) 289:2663–72. doi: 10.1001/jama.289.20.2663 12771113

[73] Espeland MA Rapp SR Shumaker SA Brunner R Manson JE Sherwin BB . Conjugated equine estrogens and global cognitive function in postmenopausal women: Women’s Health Initiative Memory Study. JAMA. (2004) 291:2959–68. doi: 10.1001/jama.291.24.2959 15213207

[74] Dunkin J Rasgon N Wagner-Steh K David S Altshuler L Rapkin A . Reproductive events modify the effects of estrogen replacement therapy on cognition in healthy postmenopausal women. Psychoneuroendocrinology. (2005) 30:284–96. doi: 10.1016/j.psyneuen.2004.09.002 15511602

[75] Greenspan SL Beck TJ Resnick NM Bhattacharya R Parker RA . Effect of hormone replacement, alendronate, or combination therapy on hip structural geometry: a 3-year, double-blind, placebo-controlled clinical trial. J Bone Miner Res. (2005) 20:1525–32. doi: 10.1359/JBMR.050508 16059624

[76] Schiff R Bulpitt CJ Wesnes KA Rajkumar C . Short-term transdermal estradiol therapy, cognition and depressive symptoms in healthy older women. A randomised placebo controlled pilot cross-over study. Psychoneuroendocrinology. (2005) 30:309–15. doi: 10.1016/j.psyneuen.2004.08.007 15694110

[77] Wolf OT Heinrich AB Hanstein B Kirschbaum C . Estradiol or estradiol/progesterone treatment in older women: no strong effects on cognition. Neurobiol Aging. (2005) 26:1029–33. doi: 10.1016/j.neurobiolaging.2004.09.012 15748783

[78] Almeida OP Lautenschlager NT Vasikaran S Leedman P Gelavis A Flicker L . A 20-week randomized controlled trial of estradiol replacement therapy for women aged 70 years and older: effect on mood, cognition and quality of life. Neurobiol Aging. (2006) 27:141–9. doi: 10.1016/j.neurobiolaging.2004.12.012 16298249

[79] Resnick SM Maki PM Rapp SR Espeland MA Brunner R Coker LH . Effects of combination estrogen plus progestin hormone treatment on cognition and affect. J Clin Endocrinol Metab. (2006) 91:1802–10. doi: 10.1210/jc.2005-2097 16522699

[80] Yaffe K Vittinghoff E Ensrud KE Johnson KC Diem S Hanes V . Effects of ultra-low-dose transdermal estradiol on cognition and health-related quality of life. Arch Neurol. (2006) 63:945–50. doi: 10.1001/archneur.63.7.945 16831962

[81] Maki PM Gast MJ Vieweg AJ Burriss SW Yaffe K . Hormone therapy in menopausal women with cognitive complaints: a randomized, double-blind trial. Neurology. (2007) 69:1322–30. doi: 10.1212/01.wnl.0000277275.42504.93 17893293

[82] Pefanco MA Kenny AM Kaplan RF Kuchel G Walsh S Kleppinger A . The effect of 3-year treatment with 0.25 mg/day of micronized 17beta-estradiol on cognitive function in older postmenopausal women. J Am Geriatr Soc. (2007) 55:426–31. doi: 10.1111/j.1532-5415.2007.01085.x 17341247

[83] Marinho RM Soares JM Jr. Santiago RC Maganhin CC MaChado F De Miranda Cota AM . Effects of estradiol on the cognitive function of postmenopausal women. Maturitas. (2008) 60:230–4. doi: 10.1016/j.maturitas.2008.07.003 18775608

[84] Resnick SM Espeland MA An Y Maki PM Coker LH Jackson R . Effects of conjugated equine estrogens on cognition and affect in postmenopausal women with prior hysterectomy. J Clin Endocrinol Metab. (2009) 94:4152–61. doi: 10.1210/jc.2009-1340 PMC2775644 19850684

[85] Tierney MC Oh P Moineddin R Greenblatt EM Snow WG Fisher RH . A randomized double-blind trial of the effects of hormone therapy on delayed verbal recall in older women. Psychoneuroendocrinology. (2009) 34:1065–74. doi: 10.1016/j.psyneuen.2009.02.009 19297102

[86] Alhola P Tuomisto H Saarinen R Portin R Kalleinen N Polo-Kantola P . Estrogen + progestin therapy and cognition: a randomized placebo-controlled double-blind study. J Obstet Gynaecol Res. (2010) 36:796–802. doi: 10.1111/j.1447-0756.2010.01214.x 20666948

[87] Kocoska-Maras L Zethraeus N Radestad AF Ellingsen T Von Schoultz B Johannesson M . A randomized trial of the effect of testosterone and estrogen on verbal fluency, verbal memory, and spatial ability in healthy postmenopausal women. Fertil Steril. (2011) 95:152–7. doi: 10.1016/j.fertnstert.2010.05.062 20667535

[88] Davison SL Bell RJ Robinson PJ Jane F Leech J Maruff P . Continuous-combined oral estradiol/drospirenone has no detrimental effect on cognitive performance and improves estrogen deficiency symptoms in early postmenopausal women: a randomized placebo-controlled trial. Menopause. (2013) 20:1020–6. doi: 10.1097/GME.0b013e318287474f 23591255

[89] Berent-Spillson A Briceno E Pinsky A Simmen A Persad CC Zubieta JK . Distinct cognitive effects of estrogen and progesterone in menopausal women. Psychoneuroendocrinology. (2015) 59:25–36. doi: 10.1016/j.psyneuen.2015.04.020 26010861 PMC4490102

[90] Jayachandran M Miller VM Lahr BD Bailey KR Lowe VJ Fields JA . Peripheral markers of neurovascular unit integrity and amyloid-β in the brains of menopausal women. J Alzheimers Dis. (2021) 80:397–405. doi: 10.3233/JAD-201410 33554914 PMC8075395

[91] Mikolajewicz N Komarova SV . Meta-analytic methodology for basic research: A practical guide. Front Physiol. (2019) 10:203. doi: 10.3389/fphys.2019.00203 30971933 PMC6445886

[92] Higgins Jpt ES Li T . Chapter 23: Including variants on randomized trials. In: Cochrane handbook for systematic reviews of interventions, 2nd edition. John Wiley & Sons, Chichester (UK (2022).

[93] Vanderweele TJ . Optimal approximate conversions of odds ratios and hazard ratios to risk ratios. Biometrics. (2020) 76:746–52. doi: 10.1111/biom.13197 31808145

[94] Higgins JP Thompson SG . Quantifying heterogeneity in a meta-analysis. Stat Med. (2002) 21:1539–58. doi: 10.1002/sim.1186 12111919

[95] Hedges LV Olkin I . Statistical methods for meta-analysis. Orlando: Academic Press (1985).

[96] Dersimonian R Laird N . Meta-analysis in clinical trials. Control Clin Trials. (1986) 7:177–88. doi: 10.1016/0197-2456(86)90046-2 3802833

[97] Jackson D White IR Thompson SG . Extending DerSimonian and Laird’s methodology to perform multivariate random effects meta-analyses. Stat Med. (2010) 29:1282–97. doi: 10.1002/sim.3602 19408255

[98] Leblanc ES Janowsky J Chan BK Nelson HD . Hormone replacement therapy and cognition: systematic review and meta-analysis. JAMA. (2001) 285:1489–99. doi: 10.1001/jama.285.11.1489 11255426

[99] Yesufu A Bandelow S Hogervorst E . Meta-analyses of the effect of hormone treatment on cognitive function in postmenopausal women. Womens Health (Lond). (2007) 3:173–94. doi: 10.2217/17455057.3.2.173 19803851

[100] Lethaby A Hogervorst E Richards M Yesufu A Yaffe K . Hormone replacement therapy for cognitive function in postmenopausal women. Cochrane Database Syst Rev. (2008) 2008(1):CD003122. doi: 10.1002/14651858.CD003122.pub2 18254016 PMC6599876

[101] Hogervorst E Bandelow S . Sex steroids to maintain cognitive function in women after the menopause: a meta-analyses of treatment trials. Maturitas. (2010) 66:56–71. doi: 10.1016/j.maturitas.2010.02.005 20202765

[102] Maki PM Zonderman AB Resnick SM . Enhanced verbal memory in nondemented elderly women receiving hormone-replacement therapy. Am J Psychiatry. (2001) 158:227–33. doi: 10.1176/appi.ajp.158.2.227 11156805

[103] Ockene JK Barad DH Cochrane BB Larson JC Gass M Wassertheil-Smoller S . Symptom experience after discontinuing use of estrogen plus progestin. JAMA. (2005) 294:183–93. doi: 10.1001/jama.294.2.183 16014592

[104] Cheung MW . Modeling dependent effect sizes with three-level meta-analyses: a structural equation modeling approach. Psychol Methods. (2014) 19:211–29. doi: 10.1037/a0032968 23834422

[105] Egger M Davey Smith G Schneider M Minder C . Bias in meta-analysis detected by a simple, graphical test. BMJ. (1997) 315:629–34. doi: 10.1136/bmj.315.7109.629 PMC2127453 9310563

[106] Duval S Tweedie R . Trim and fill: A simple funnel-plot-based method of testing and adjusting for publication bias in meta-analysis. Biometrics. (2000) 56:455–63. doi: 10.1111/j.0006-341X.2000.00455.x 10877304

[107] Zhou HH Yu Z Luo L Xie F Wang Y Wan Z . The effect of hormone replacement therapy on cognitive function in healthy postmenopausal women: a meta-analysis of 23 randomized controlled trials. Psychogeriatrics. (2021) 21:926–38. doi: 10.1111/psyg.12768 34622524

[108] Chen L Zheng W Chen G Liu LH Yao J Chen Y . Menopausal hormone therapy does not improve some domains of memory: A systematic review and meta-analysis. Front Endocrinol (Lausanne). (2022) 13:894883. doi: 10.3389/fendo.2022.894883 36147572 PMC9486389

[109] Rocca WA Bower JH Maraganore DM Ahlskog JE Grossardt BR De Andrade M . Increased risk of cognitive impairment or dementia in women who underwent oophorectomy before menopause. Neurology. (2007) 69:1074–83. doi: 10.1212/01.wnl.0000276984.19542.e6 17761551

[110] Phung TK Waltoft BL Laursen TM Settnes A Kessing LV Mortensen PB . Hysterectomy, oophorectomy and risk of dementia: a nationwide historical cohort study. Dement Geriatr Cognit Disord. (2010) 30:43–50. doi: 10.1159/000314681 20689282

[111] Rocca WA Grossardt BR Shuster LT . Oophorectomy, estrogen, and dementia: a 2014 update. Mol Cell Endocrinol. (2014) 389:7–12. doi: 10.1016/j.mce.2014.01.020 24508665 PMC4040304

[112] Gilsanz P Lee C Corrada MM Kawas CH Quesenberry CP Jr. Whitmer RA . Reproductive period and risk of dementia in a diverse cohort of health care members. Neurology. (2019) 92:e2005–14. doi: 10.1212/WNL.0000000000007326 PMC6511081 30923235

[113] Imtiaz B Tuppurainen M Tiihonen M Kivipelto M Soininen H Hartikainen S . Oophorectomy, hysterectomy, and risk of Alzheimer’s disease: a nationwide case-control study. J Alzheimers Dis. (2014) 42:575–81. doi: 10.3233/JAD-140336 24898656

[114] Hackman BW Galbraith D . Replacement therapy and piperazine oestrone sulphate (‘Harmogen’) and its effect on memory. Curr Med Res Opin. (1976) 4:303–6. 10.1185/03007997609109322 791587

[115] Sherwin BB . Estrogen and/or androgen replacement therapy and cognitive functioning in surgically menopausal women. Psychoneuroendocrinology. (1988) 13:345–57. doi: 10.1016/0306-4530(88)90060-1 3067252

[116] Sherwin BB . Estrogen and cognitive functioning in surgically menopausal women. Ann NY Acad Sci. (1990) 592:474–5. doi: 10.1111/j.1749-6632.1990.tb30379.x

[117] Matyi JM Rattinger GB Schwartz S Buhusi M Tschanz JT . Lifetime estrogen exposure and cognition in late life: the Cache County Study. Menopause. (2019) 26:1366–74. doi: 10.1097/GME.0000000000001405 PMC7448538 31613825

[118] Kampen DL Sherwin BB . Estrogen use and verbal memory in healthy postmenopausal women. Obstet Gynecol. (1994) 83:979–83. doi: 10.1097/00006250-199406000-00017 8190445

[119] Robinson D Friedman L Marcus R Tinklenberg J Yesavage J . Estrogen replacement therapy and memory in older women. J Am Geriatr Soc. (1994) 42:919–22. doi: 10.1111/j.1532-5415.1994.tb06580.x 8064097

[120] Kimura D . Estrogen replacement therapy may protect against intellectual decline in postmenopausal women. Hormones Behav. (1995) 29:312–21. doi: 10.1006/hbeh.1995.1022 7490007

[121] Szklo M Cerhan J Diez-Roux AV Chambless L Cooper L Folsom AR . Estrogen replacement therapy and cognitive functioning in the Atherosclerosis Risk in Communities (ARIC) Study. Am J Epidemiol. (1996) 144:1048–57. doi: 10.1093/oxfordjournals.aje.a008877 8942436

[122] Resnick SM Maki PM Golski S Kraut MA Zonderman AB . Effects of estrogen replacement therapy on PET cerebral blood flow and neuropsychological performance. Hormones Behav. (1998) 34:171–82. doi: 10.1006/hbeh.1998.1476 9799627

[123] Maki PM . A systematic review of clinical trials of hormone therapy on cognitive function: effects of age at initiation and progestin use. Ann N Y Acad Sci. (2005) 1052:182–97. doi: 10.1196/annals.1347.012 16024761

[124] Greendale GA Huang MH Wight RG Seeman T Luetters C Avis NE . Effects of the menopause transition and hormone use on cognitive performance in midlife women. Neurology. (2009) 72:1850–7. doi: 10.1212/WNL.0b013e3181a71193 PMC2690984 19470968

[125] Maki PM Henderson VW . Cognition and the menopause transition. Menopause. (2016) 23:803–5. doi: 10.1097/GME.0000000000000681 27272226

[126] Woods NF Mitchell ES Adams C . Memory functioning among midlife women: observations from the Seattle Midlife Women’s Health Study. Menopause. (2000) 7:257–65. doi: 10.1097/00042192-200007040-00008 10914619

[127] Buckley RF O’donnell A Mcgrath ER Jacobs HIL Lois C Satizabal CL . Menopause status moderates sex differences in tau burden: A framingham PET study. Ann Neurol. (2022) 92:11–22. doi: 10.1002/ana.26382 35471588 PMC9233144

[128] Mosconi L Berti V Quinn C Mchugh P Petrongolo G Osorio RS . Correction: Perimenopause and emergence of an Alzheimer’s bioenergetic phenotype in brain and periphery. PloS One. (2018) 13:e0193314. doi: 10.1371/journal.pone.0193314 29447296 PMC5814030

[129] Kim GW Park K Jeong GW . Effects of sex hormones and age on brain volume in post-menopausal women. J Sex Med. (2018) 15:662–70. doi: 10.1016/j.jsxm.2018.03.006 29628218

[130] Schelbaum E Loughlin L Jett S Zhang C Jang G Malviya N . Association of reproductive history with brain MRI biomarkers of dementia risk in midlife. Neurology. (2021) 97:e2328–39. doi: 10.1212/WNL.0000000000012941 PMC8665431 34732544

[131] Zeydan B Tosakulwong N Schwarz CG Senjem ML Gunter JL Reid RI . Association of bilateral salpingo-oophorectomy before menopause onset with medial temporal lobe neurodegeneration. JAMA Neurol. (2019) 76:95–100. doi: 10.1001/jamaneurol.2018.3057 30326011 PMC6439881

[132] Jett S Dyke JP Andy C Schelbaum E Jang G Boneu Yepez C . Effects of sex and APOE ϵ4 genotype on brain mitochondrial high-energy phosphates in midlife individuals at risk for Alzheimer’s disease: a 31Phosphorus MR spectroscopy study. PloS One. (2023) 18(2):e0281302. doi: 10.1371/journal.pone.0281302 36787293 PMC9928085

[133] Jett S Dyke JP Andy C Schelbaum E Jang G Boneu Yepez C . Sex and menopause impact (31)P-Magnetic Resonance Spectroscopy brain mitochondrial function in association with (11)C-PiB PET amyloid-beta load. Sci Rep. (2022) 12:22087. doi: 10.1038/s41598-022-26573-5 36543814 PMC9772209

[134] Lohner V Pehlivan G Sanroma G Miloschewski A Schirmer MD Stocker T . Relation between sex, menopause, and white matter hyperintensities: the rhineland study. Neurology. (2022) 99:e935–43. doi: 10.1212/WNL.0000000000200782 PMC9502737 35768207

[135] Eberling JL Reed BR Coleman JE Jagust WJ . Effect of estrogen on cerebral glucose metabolism in postmenopausal women. Neurology. (2000) 55:875–7. doi: 10.1212/WNL.55.6.875 10994014

[136] Rasgon NL Silverman D Siddarth P Miller K Ercoli LM Elman S . Estrogen use and brain metabolic change in postmenopausal women. Neurobiol Aging. (2005) 26:229–35. doi: 10.1016/j.neurobiolaging.2004.03.003 15582750

[137] Silverman DH Geist CL Kenna HA Williams K Wroolie T Powers B . Differences in regional brain metabolism associated with specific formulations of hormone therapy in postmenopausal women at risk for AD. Psychoneuroendocrinology. (2011) 36:502–13. doi: 10.1016/j.psyneuen.2010.08.002 PMC3021636 20810219

[138] Rasgon NL Geist CL Kenna HA Wroolie TE Williams KE Silverman DH . Prospective randomized trial to assess effects of continuing hormone therapy on cerebral function in postmenopausal women at risk for dementia. PloS One. (2014) 9:e89095. doi: 10.1371/journal.pone.0089095 24622517 PMC3951184

[139] Maki PM Resnick SM . Longitudinal effects of estrogen replacement therapy on PET cerebral blood flow and cognition. Neurobiol Aging. (2000) 21:373–83. doi: 10.1016/S0197-4580(00)00123-8 10867223

[140] Slopien R Junik R Meczekalski B Halerz-Nowakowska B Maciejewska M Warenik-Szymankiewicz A . Influence of hormonal replacement therapy on the regional cerebral blood flow in postmenopausal women. Maturitas. (2003) 46:255–62. doi: 10.1016/S0378-5122(03)00144-0 14625122

[141] Kantarci K Lowe VJ Lesnick TG Tosakulwong N Bailey KR Fields JA . Early postmenopausal transdermal 17β-estradiol therapy and amyloid-β Deposition. J Alzheimers Dis. (2016) 53:547–56. doi: 10.3233/JAD-160258 PMC4955514 27163830

[142] Wisch JK Meeker KL Gordon BA Flores S Dincer A Grant EA . Sex-related differences in tau positron emission tomography (PET) and the effects of hormone therapy (HT). Alzheimer Dis Assoc Disord. (2021) 35:164–8. doi: 10.1097/WAD.0000000000000393 PMC7725985 32520734

[143] Depypere H Vergallo A Lemercier P Lista S Benedet A Ashton N . Menopause hormone therapy significantly alters pathophysiological biomarkers of Alzheimer’s disease. Alzheimers Dement. (2023) 19(4):1320–30. doi: 10.1002/alz.12759 36218064

[144] Nilsen J Brinton RD . Divergent impact of progesterone and medroxyprogesterone acetate (Provera) on nuclear mitogen-activated protein kinase signaling. Proc Natl Acad Sci U.S.A. (2003) 100:10506–11. doi: 10.1073/pnas.1334098100 PMC193591 12925744

[145] Nilsen J Brinton RD . Impact of progestins on estradiol potentiation of the glutamate calcium response. Neuroreport. (2002) 13:825–30. doi: 10.1097/00001756-200205070-00018 11997695

[146] Kim Y . Precision hormone therapy: gaps and opportunities. Gynecol Reprod Endocrinol Metab. (2020) 1:80–8.

