# Supplementary material for: Systematic review and meta-analysis of the effects of menopause hormone therapy on cognition
Source: Front Endocrinol (Lausanne). 2024 Mar 4;15:1350318. doi: 10.3389/fendo.2024.1350318 (PMC10944893; doi:10.3389/fendo.2024.1350318)
Supplement: Supplementary file 1 [file Image_1.pdf]

e-Figure 1. Meta-analysis of MHT effects on global cognition

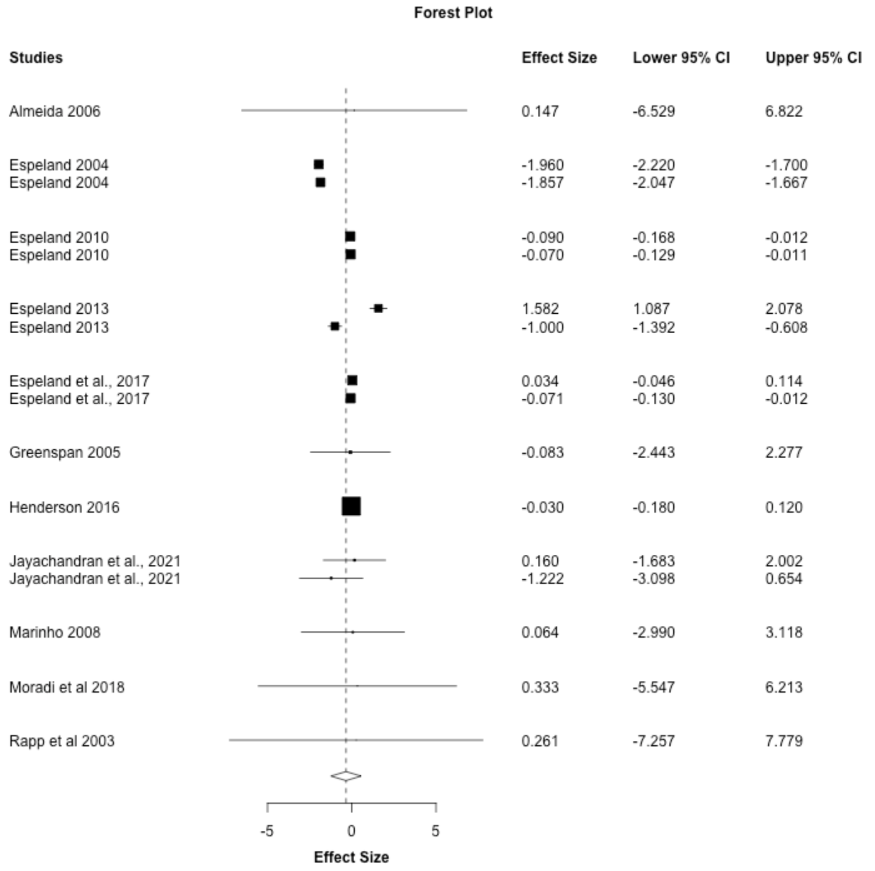

Meta-analysis of randomized placebo-controlled trials investigating the effects of systemic menopause hormone therapy (MHT) on global cognition. As several studies include multiple effect estimates for different exposure types, as well as multiple outcome measures, we applied Robust Variance Estimation (RVE) to compute the pooled effect size (VanderWeele, 2020). RVE accounts for intra-study dependent effect sizes while mitigating the impact of outliers, unequal variances, and other sources of heterogeneity (VanderWeele, 2020). Forest plots display individual and pooled estimates of the association between MHT use and global cognition scores expressed as effect size (standardized mean difference, SMD) and 95% confidence intervals (C.I.). Studies are displayed in alphabetical order.

e-Figure 2. Meta-analysis of MHT effects on verbal memory

Forest Plot

Studies	Effect Size	Lower 95% CI	Upper 95% CI
Alhola 2010	1.261	-2.471	4.992
Alhola 2010	0.158	-3.561	3.877
Almeida 2006	-0.130	-16.757	16.497
Almeida 2006	0.000	-4.811	4.811
Berent-Spillson 2015,	0.326	-6.883	7.535
Berent-Spillson 2015,	0.249	-2.898	3.396
Binder et al 2001	-0.299	-6.208	5.611
Binder et al 2001	0.000	-1.960	1.960
Davison et al 2013	1.327	-0.150	2.804
Dunkin et al 2005	0.000	-1.960	1.960
Dunkin et al 2005	-3.000	-4.960	-1.040
Dunkin et al 2005	0.000	-3.036	3.036
Dunkin et al 2005	0.000	-5.880	5.880
Dunkin et al 2005	2.500	-5.340	10.340
Dunkin et al 2005	0.000	-1.960	1.960
Espeland 2010	-0.040	-0.158	0.078
Espeland 2010	-0.020	-0.098	0.058
Espeland 2013	0.000	-0.176	0.176
Espeland 2013	0.000	-0.114	0.114
Espeland 2013	0.000	-0.137	0.137
Espeland 2013	1.250	1.093	1.407
Espeland et al., 2017	-0.035	-0.094	0.024
Espeland et al., 2017	-0.023	-0.082	0.036
Espeland et al., 2017	-0.020	-0.059	0.019
Espeland et al., 2017	-0.050	-0.109	0.009
Gorenstein 2011	0.000	-5.880	5.880
Gorenstein 2011	0.000	-5.016	5.016
Gorenstein 2011	0.000	-5.880	5.880
Henderson 2016	-0.060	-0.220	0.100
Joffe 2006	-1.249	-4.387	1.888
Joffe 2006	0.980	-0.621	2.580
Joffe 2006	1.281	-1.779	4.341
Kocoska-Maras et al., 2011	0.000	-10.484	10.484
LeBlanc 2007	1.000	-0.960	2.960
LeBlanc 2007	0.000	-1.960	1.960
LeBlanc 2007	0.659	-2.313	3.632
LeBlanc 2007	0.000	-1.486	1.486
Linzmayer 2001	2.000	0.040	3.960
Linzmayer 2001	0.500	-3.420	4.420
Linzmayer 2001	0.000	-3.129	3.129
Linzmayer 2001	0.000	-3.920	3.920
Linzmayer 2001	-0.243	-8.324	7.839
Linzmayer 2001	0.000	-3.099	3.099
Maki 2007	-0.228	-5.640	5.184
Maki 2007	-0.011	-3.679	3.658
Maki 2007	0.051	-7.593	7.695
Maki 2007	0.127	-7.616	7.869
Maki et al 2009	-0.622	-11.778	10.534
Maki et al 2009	-0.283	-7.212	6.647
Maki et al 2009	0.170	-7.196	7.537
Maki et al 2009	-0.025	-7.865	7.815
Marinho 2008	0.166	-11.664	11.995
Marinho 2008	0.182	-10.596	10.960
Pefanco 2007	-4.000	-5.960	-2.040
Pefanco 2007	0.650	-2.367	3.666
Phillips & Sherwin 1992	0.397	-4.543	5.337
Phillips & Sherwin 1992	0.000	-4.940	4.940
Phillips & Sherwin 1992	3.864	0.820	6.907
Phillips & Sherwin 1992	1.000	-0.960	2.960
Resnick 2006	0.333	-5.547	6.213
Resnick 2006	0.000	-5.880	5.880
Resnick 2009	-1.143	-1.280	-1.006
Resnick 2009	-1.143	-1.280	-1.006
Schiff 2005	-0.159	-24.860	24.542
Schiff 2005	-0.080	-24.657	24.497
Tierney 2009	0.762	-1.040	2.563
Wolf 1999	-3.000	-4.960	-1.040
Wolf 1999	-3.000	-4.960	-1.040
Wolf 2005	-1.933	-2.440	-1.426
Wolf 2005	0.500	-1.460	2.460
Wolf 2005	1.000	-0.960	2.960
Wolf 2005	-2.000	-5.920	1.920
Wolf 2005	-2.633	-3.377	-1.888
Wolf 2005	-1.000	-2.960	0.960
Wolf 2005	-1.000	-2.960	0.960
Wolf 2005	-1.000	-4.920	2.920
Yaffe 2006	-0.094	-7.370	7.182
Yaffe 2006	-0.051	-7.698	7.595
Yaffe 2006	0.032	-6.047	6.111
Yaffe 2006	-0.020	-2.960	2.920

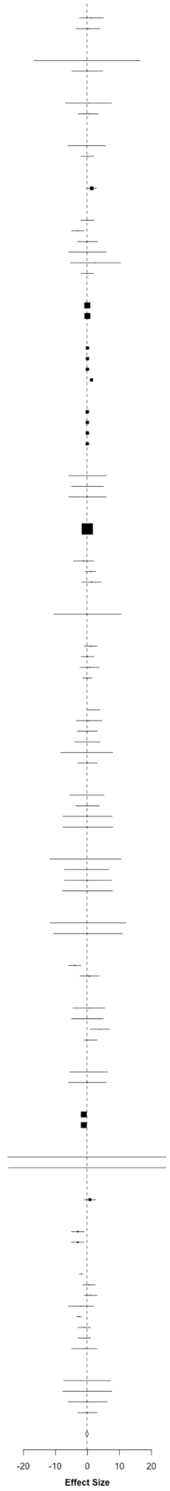

Meta-analysis of randomized placebo-controlled trials investigating the effects of systemic menopause hormone therapy (MHT) on verbal memory. As several studies include multiple effect estimates for different exposure types, as well as multiple outcome measures, we applied Robust Variance Estimation (RVE) to compute the pooled effect size (VanderWeele, 2020). RVE accounts for intra-study dependent effect sizes while mitigating the impact of outliers, unequal variances, and other sources of heterogeneity (VanderWeele, 2020). Forest plots display individual and pooled estimates of the association between MHT use and global cognition scores expressed as effect size (standardized mean difference, SMD) and 95% confidence intervals (C.I.). Studies are displayed in alphabetical order.

e-Figure 3. Meta-analysis of MHT effects on visual memory

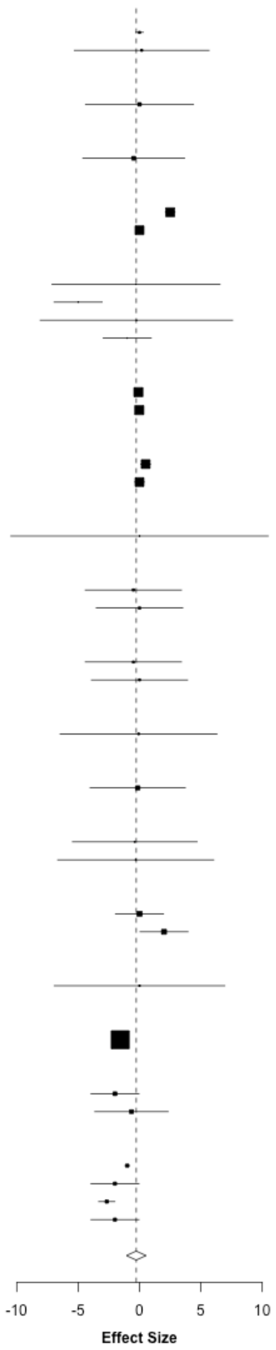

Studies	Effect Size	Lower 95% CI	Upper 95% CI
Alhola 2010	0.000	-0.310	0.310
Alhola 2010	0.178	-5.313	5.670
Berent-Spillson 2015,	0.000	-4.400	4.400
Davison et al 2013	-0.472	-4.621	3.676
Duka 2000	2.500	2.108	2.892
Duka 2000	0.000	-0.307	0.307
Dunkin et al 2005	-0.286	-7.132	6.560
Dunkin et al 2005	-5.000	-6.960	-3.040
Dunkin et al 2005	-0.250	-8.090	7.590
Dunkin et al 2005	-1.000	-2.960	0.960
Espeland 2010	-0.090	-0.168	-0.012
Espeland 2010	-0.020	-0.079	0.039
Joffe 2006	0.500	0.108	0.892
Joffe 2006	0.000	-0.392	0.392
Kocoska-Maras et al., 2011	0.000	-10.484	10.484
LeBlanc 2007	-0.500	-4.420	3.420
LeBlanc 2007	0.000	-3.529	3.529
Linzmayer 2001	-0.500	-4.420	3.420
Linzmayer 2001	0.000	-3.920	3.920
Maki 2007	-0.077	-6.468	6.314
Maki et al 2009	-0.146	-4.029	3.737
Pefanco 2007	-0.386	-5.466	4.695
Pefanco 2007	-0.308	-6.667	6.051
Phillips & Sherwin 1992	0.000	-1.960	1.960
Phillips & Sherwin 1992	2.000	0.040	3.960
Resnick 2006	0.000	-6.950	6.950
Resnick 2009	-1.571	-1.709	-1.434
Wolf 1999	-2.000	-3.960	-0.040
Wolf 1999	-0.655	-3.649	2.339
Wolf 2005	-1.000	-1.196	-0.804
Wolf 2005	-2.000	-3.960	-0.040
Wolf 2005	-2.668	-3.330	-2.007
Wolf 2005	-2.000	-3.960	-0.040

Meta-analysis of randomized placebo-controlled trials investigating the effects of systemic menopause hormone therapy (MHT) on visual memory. As several studies include multiple

effect estimates for different exposure types, as well as multiple outcome measures, we applied Robust Variance Estimation (RVE) to compute the pooled effect size (VanderWeele, 2020). RVE accounts for intra-study dependent effect sizes while mitigating the impact of outliers, unequal variances, and other sources of heterogeneity (VanderWeele, 2020). Forest plots display individual and pooled estimates of the association between MHT use and global cognition scores expressed as effect size (standardized mean difference, SMD) and 95% confidence intervals (C.I.). Studies are displayed in alphabetical order.

e-Figure 4. Meta-analysis of MHT effects on visuospatial performance

Studies	Effect Size	Lower 95% CI	Upper 95% CI
Alhola 2010	-0.316	-8.996	8.363
Almeida 2006	0.368	-10.820	11.555
Dunkin et al 2005	-0.469	-2.141	1.204
Espeland 2010	-0.020	-0.138	0.098
Espeland 2010	0.020	-0.058	0.098
Joffe 2006	1.000	-0.960	2.960
Maki 2007	0.073	-56.684	56.829
Maki et al 2009	0.322	-28.249	28.894
Marinho 2008	1.027	-14.245	16.299
Pefanco 2007	0.500	-3.420	4.420
Resnick 2006	-0.036	-54.916	54.844
Resnick 2009	2.625	1.684	3.566
Wolf 1999	-2.000	-3.960	-0.040

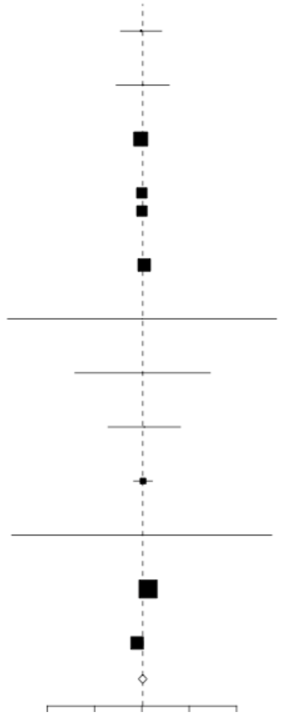

Meta-analysis of randomized placebo-controlled trials investigating the effects of systemic menopause hormone therapy (MHT) on visuospatial performance. As several studies include multiple effect estimates for different exposure types, as well as multiple outcome measures, we applied Robust Variance Estimation (RVE) to compute the pooled effect size (VanderWeele, 2020). RVE accounts for intra-study dependent effect sizes while mitigating the impact of outliers, unequal variances, and other sources of heterogeneity (VanderWeele, 2020). Forest plots display individual and pooled estimates of the association between MHT use and global cognition scores expressed as effect size (standardized mean difference, SMD) and 95% confidence intervals (C.I.). Studies are displayed in alphabetical order.

e-Figure 5. Meta-analysis of MHT effects on working memory

Forest Plot

Studies	Effect Size	Lower 95% CI	Upper 95% CI
Alhola 2010	-0.527	-19.121	18.067
Alhola 2010	-0.130	-4.643	4.382
Dunkin et al 2005	-0.478	-8.685	7.729
Dunkin et al 2005	-0.840	-19.497	17.816
Espeland 2013	0.714	0.440	0.989
Espeland 2013	1.381	1.097	1.665
Espeland 2013	-2.611	-2.836	-2.386
Espeland 2013	0.000	-0.225	0.225
Espeland et al., 2017	0.020	-0.058	0.098
Espeland et al., 2017	-0.040	-0.099	0.019
Gorenstein 2011	0.000	-1.960	1.960
Gorenstein 2011	1.000	-0.960	2.960
Gorenstein 2011	-0.074	-26.571	26.423
Joffe 2006	0.000	-6.889	6.889
Maki 2007	0.076	-4.336	4.487
Maki 2007	-0.204	-4.047	3.639
Maki et al 2009	-0.754	-4.392	2.884
Maki et al 2009	0.416	-3.354	4.186
Marinho 2008	-0.500	-4.420	3.420
Marinho 2008	0.400	-19.200	20.000
Phillips & Sherwin 1992	-2.500	-3.284	-1.716
Resnick 2006	0.000	-3.920	3.920
Resnick 2006	0.000	-3.920	3.920
Resnick 2009	0.750	0.672	0.828
Resnick 2009	-1.250	-1.328	-1.172
Wolf 2005	1.000	-0.960	2.960
Wolf 2005	1.408	0.016	2.800
Wolf 2005	1.000	-0.960	2.960
Wolf 2005	0.000	-0.928	0.928

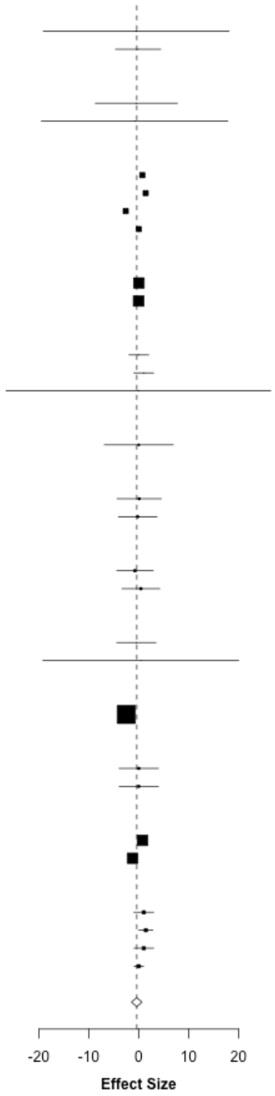

Meta-analysis of randomized placebo-controlled trials investigating the effects of systemic menopause hormone therapy (MHT) on working memory. As several studies include multiple effect estimates for different exposure types, as well as multiple outcome measures, we applied Robust Variance Estimation (RVE) to compute the pooled effect size (VanderWeele, 2020). RVE accounts for intra-study dependent effect sizes while mitigating the impact of outliers, unequal variances, and other sources of heterogeneity (VanderWeele, 2020). Forest plots display individual and pooled estimates of the association between MHT use and global cognition scores expressed as effect size (standardized mean difference, SMD) and 95% confidence intervals (C.I.). Studies are displayed in alphabetical order.

e-Figure 6. Meta-analysis of MHT effects on fluency

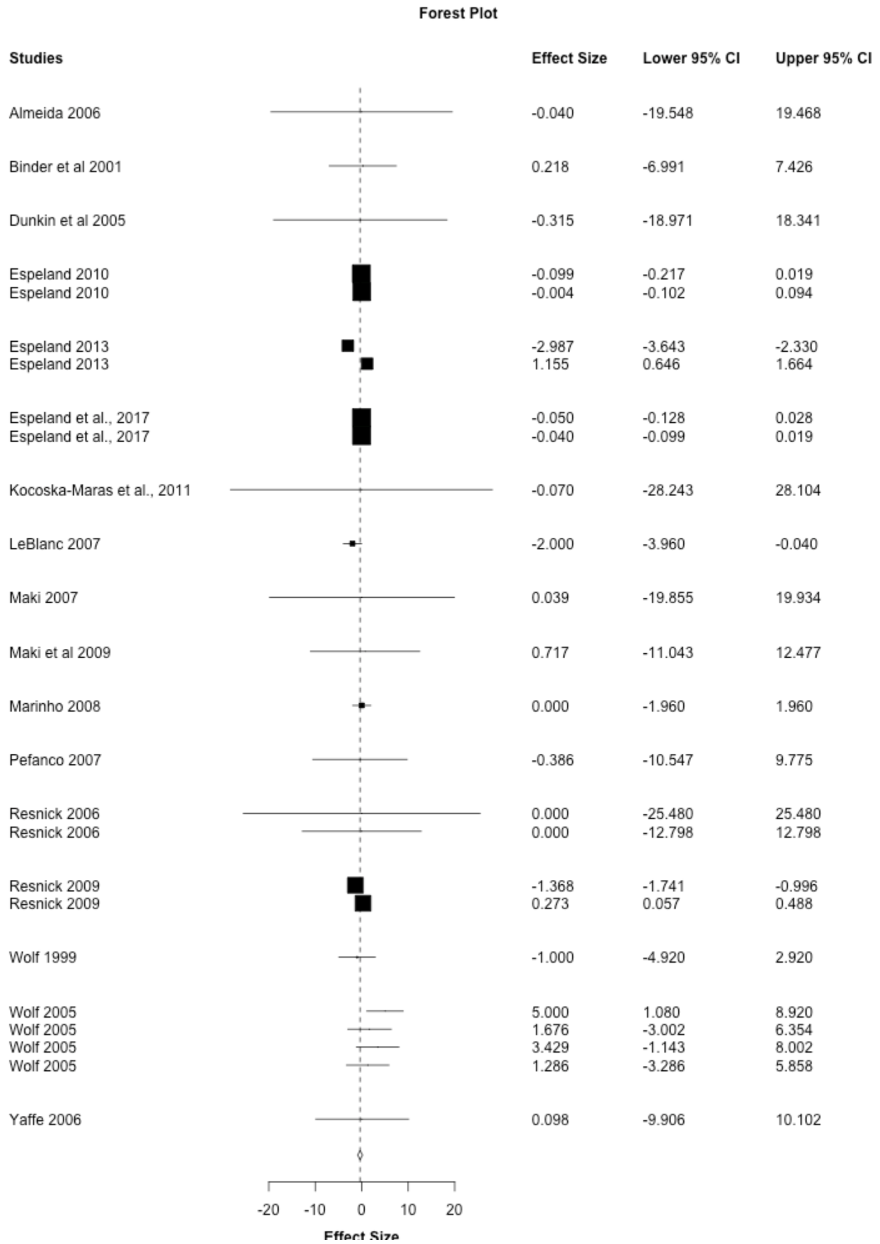

Meta-analysis of randomized placebo-controlled trials investigating the effects of systemic menopause hormone therapy (MHT) on fluency. As several studies include multiple effect estimates for different exposure types, as well as multiple outcome measures, we applied Robust Variance Estimation (RVE) to compute the pooled effect size (VanderWeele, 2020). RVE accounts for intra-study dependent effect sizes while mitigating the impact of outliers, unequal variances, and other sources of heterogeneity (VanderWeele, 2020). Forest plots display individual and pooled estimates of the association between MHT use and global cognition scores expressed as effect size (standardized mean difference, SMD) and 95% confidence intervals (C.I.). Studies are displayed in alphabetical order.

e-Figure 7. Meta-analysis of MHT effects on executive function

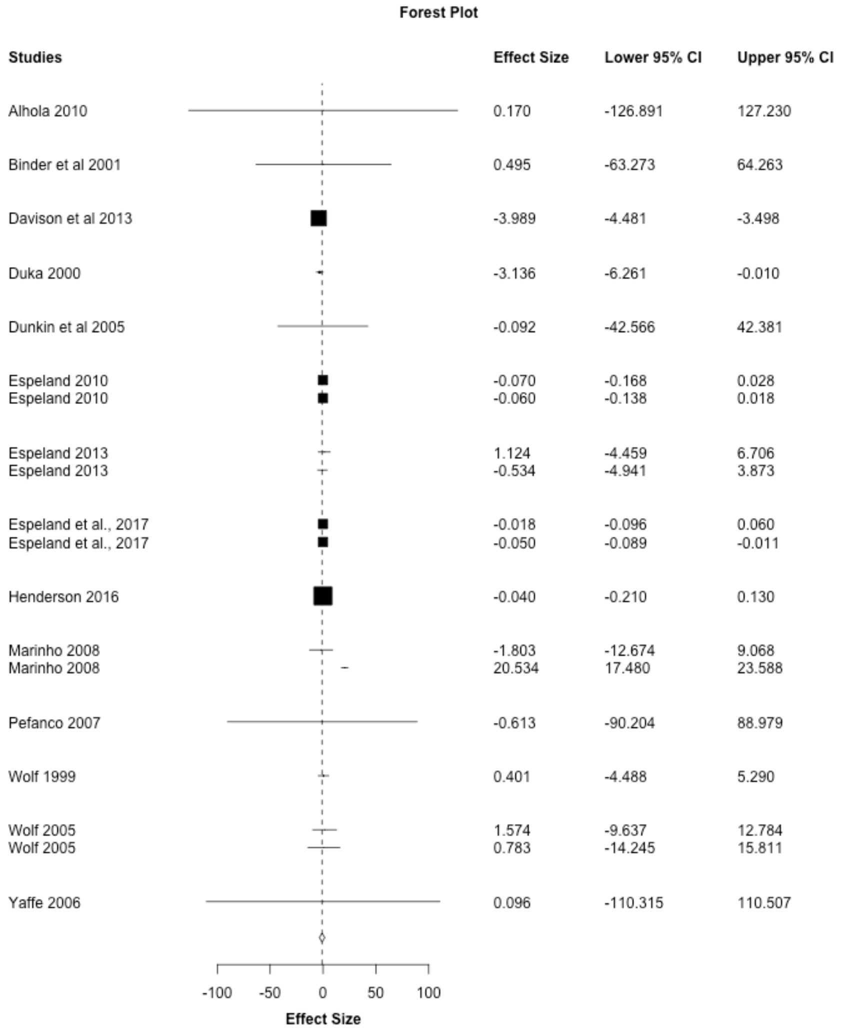

Meta-analysis of randomized placebo-controlled trials investigating the effects of systemic menopause hormone therapy (MHT) on executive function. As several studies include multiple effect estimates for different exposure types, as well as multiple outcome measures, we applied Robust Variance Estimation (RVE) to compute the pooled effect size (VanderWeele, 2020). RVE accounts for intra-study dependent effect sizes while mitigating the impact of outliers, unequal variances, and other sources of heterogeneity (VanderWeele, 2020). Forest plots display individual and pooled estimates of the association between MHT use and global cognition scores expressed as effect size (standardized mean difference, SMD) and 95% confidence intervals (C.I.). Studies are displayed in alphabetical order.

e-Figure 8. Meta-analysis of MHT effects on language

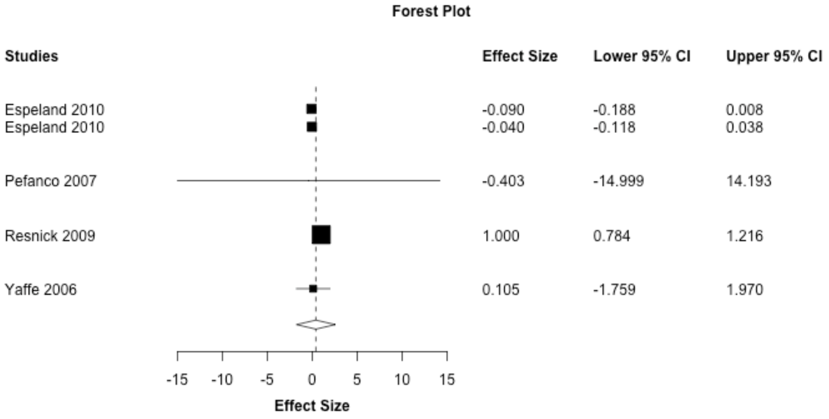

Meta-analysis of randomized placebo-controlled trials investigating the effects of systemic menopause hormone therapy (MHT) on executive function. As several studies include multiple effect estimates for different exposure types, as well as multiple outcome measures, we applied Robust Variance Estimation (RVE) to compute the pooled effect size (VanderWeele, 2020). RVE accounts for intra-study dependent effect sizes while mitigating the impact of outliers, unequal variances, and other sources of heterogeneity (VanderWeele, 2020). Forest plots display individual and pooled estimates of the association between MHT use and global cognition scores expressed as effect size (standardized mean difference, SMD) and 95% confidence intervals (C.I.). Studies are displayed in alphabetical order.
