# Supplementary material for: Systematic review and meta-analysis of the effects of menopause hormone therapy on cognition
Source: Front Endocrinol (Lausanne). 2024 Mar 4;15:1350318. doi: 10.3389/fendo.2024.1350318 (PMC10944893; doi:10.3389/fendo.2024.1350318)
Supplement: Supplementary file 2 [file Table_1.docx]

## Supplementary Table 1. Cognitive tests by domain

| Global cognition | - Cambridge Cognition Examination (CAMCOG) - Mini Mental State Exam (MMSE) - Modified MMSE (3MS) - Montreal Cognitive Assessment (MoCA) - Telephone Interview for Cognitive Status (TICS) |
| --- | --- |
| Executive function | - Groton Maze test - Oral Trail Making Test (OTMT) - Stroop test - Trail Making Test, A and B |
| Language | - Boston Naming test - Modified Boston Naming - PMA vocabulary |
| Verbal fluency | - Animals - FAS - Verbal Fluency (VF)-A |
| Verbal memory | - California Verbal Learning test (CVLT) - CDR word recall - East Boston Memory test (EBTM) - Free word recall - Grünberger Verbal Memory Test - Hopkins Verbal Learning test (HVLT) - Impact of Event Scale (IES) recall - International shopping list - Rey Auditory Verbal Learning Test (RAVLT) - Rivermead Behavioral Memory Test, Paragraph recall - Wechsler Memory Scale Logical Memory - Wechsler Memory Scale, paired associates |
| Visual memory | - Benton Visual Retention Test - BETAM delayed recall of objects - Block design test - Cambridge Neuropsychological Test Automated Battery (CANTAB) pattern recognition - CogState visual paired associates - Rey-Osterreith figure test - Wechsler Memory Scale visual reproduction |
| Visuo-spatial skills | - Bells test - Card rotation test - Figure Drawing - Mental rotation task - Wisconsin Card Sorting Test |
| Working memory | - Digit span backward - Digit span forward - Digit span total - Digit symbol substitution test |

## Supplementary Table 2. Effects of MHT on individual cognitive tests by duration of treatment

|  | **Standardized mean difference**  **(95% CI)** | **P-value** | **Heterogeneity**  **p-value** | **Publication**  **bias p-value** |
| --- | --- | --- | --- | --- |
| **Duration <1 year** | | | | |
| MMSE | NA | NA | NA | NA |
| Paired associates, immediate | 1.969 (-4.103, 8.041) | 0.273 | <0.001 | NA |
| Paired associates, delayed | 0.211 (-0.53, 0.952) | 0.418 | <0.001 | NA |
| CVLT, free recall | -0.952 (-4.976, 3.072) | 0.26 | <0.001 | NA |
| CVLT, cued recall | 0.493 (-1.833, 2.818) | 0.391 | <0.001 | NA |
| TMT-B | -1.738 (-6.575, 3.099) | 0.137 | <0.001 | NA |
| Digit span total | -2.315 (-8.106, 3.477) | 0.137 | <0.001 | NA |
| Digit span forward | -0.137 (-1.492, 1.218) | 0.605 | <0.001 | NA |
| Digit span backward | 0.692 (-1.629, 3.014) | 0.254 | <0.001 | NA |
| FAS | -0.968 (-10.931, 8.995) | 0.487 | <0.001 | NA |
| **Duration >1 year** | | | | |
| MMSE | -0.052 (-1.3, 1.196) | 0.689 | <0.001 | NA |
| Paired associates, immediate | NA | NA | NA | NA |
| Paired associates, delayed | -1.33 (-21.735, 19.074) | 0.56 | <0.001 | NA |
| CVLT, free recall | -0.456 (-7.293, 6.381) | 0.667 | <0.001 | NA |
| CVLT, cued recall | NA | NA | NA | NA |
| TMT-B | -0.018 (-0.022, -0.014) | 0.012 | <0.001 | NA |
| Digit span total | NA | NA | NA | NA |
| Digit span forward | 0.413 (-1.406, 2.233) | 0.361 | <0.001 | NA |
| Digit span backward | -0.129 (-2.603, 2.345) | 0.856 | <0.001 | NA |
| FAS | -1.349 (-4.397, 1.699) | 0.112 | <0.001 | NA |

Standardized mean difference (SMD) and associated 95% confidence interval, CI, are reported for each cognitive test, along with corresponding p-values. Positive SMDs indicate an improvement in the treated group compared to the placebo group, whereas negative SMDs indicate the opposite effect. Analyses with fewer than 10 studies are marked as not applicable (NA) for the publication bias p-values.

Abbreviations: CVLT, California Verbal Learning Test; MMSE, Mini Mental State Examination; TMT-B, Trail Making Test B.

## Supplementary Table 3. Effects of MHT on individual cognitive tests by initiation timing

|  | **Standardized mean difference**  **(95% CI)** | **P-value** | **Heterogeneity**  **p-value** | **Publication**  **bias p-value** |
| --- | --- | --- | --- | --- |
| **Midlife or before age 65** | | | | |
| MMSE | NA | NA | NA | NA |
| Paired associates, immediate | 2.249 (-8.144, 12.642) | 0.313 | <0.001 | NA |
| Paired associates, delayed | 0.267 (-1.105, 1.64) | 0.469 | <0.001 | NA |
| CVLT, free recall | -0.952 (-4.976, 3.072) | 0.260 | <0.001 | NA |
| CVLT, cued recall | 0.523 (-2.703, 3.749) | 0.413 | <0.001 | NA |
| TMT-B | NA | NA | NA | NA |
| Digit span total | -2.45 (-6.478, 1.578) | 0.083 | <0.001 | NA |
| Digit span forward | -0.03 (-0.096, 0.036) | 0.108 | <0.001 | NA |
| Digit span backward | 0.022 (-0.397, 0.441) | 0.632 | <0.001 | NA |
| FAS | -1.891 (-7.352, 3.569) | 0.150 | <0.001 | NA |
| **Midlife or after age 65** | | | | |
| MMSE | -0.052 (-1.3, 1.196) | 0.689 | <0.001 | NA |
| Paired associates, immediate | -1.48 (-11.439, 8.479) | 0.310 | <0.001 | NA |
| Paired associates, delayed | -0.945 (-5.939, 4.049) | 0.487 | <0.001 | NA |
| CVLT, free recall | -0.456 (-7.293, 6.381) | 0.667 | <0.001 | NA |
| CVLT, cued recall | NA | NA | NA | NA |
| TMT-B | -0.050 (-0.052, -0.048) | **0.002** | <0.001 | NA |
| Digit span total | NA | NA | NA | NA |
| Digit span forward | 0.399 (-1.422, 2.221) | 0.384 | <0.001 | NA |
| Digit span backward | -0.165 (-2.643, 2.313) | 0.815 | <0.001 | NA |
| FAS | -1.349 (-4.397, 1.699) | 0.112 | <0.001 | NA |

Standardized mean difference (SMD) and associated 95% confidence interval, CI, are reported for each cognitive test, along with corresponding p-values. Positive SMDs indicate an improvement in the treated group compared to the placebo group, whereas negative SMDs indicate the opposite effect. Analyses with fewer than 10 studies are marked as not applicable (NA) for the publication bias p-values.

Abbreviations: CVLT, California Verbal Learning Test; MMSE, Mini Mental State Examination; TMT-B, Trail Making Test B.

## Supplementary Table 4. Effects of MHT on individual cognitive tests by menopause type and formulation

|  | **Standardized mean difference**  **(95% CI)** | **P-value** | **Heterogeneity**  **p-value** | **Publication**  **bias p-value** |
| --- | --- | --- | --- | --- |
| **Surgical menopause** | | | | |
| MMSE | NA | NA | NA | NA |
| Paired associates, immediate | 1.067 (-36.145, 38.279) | 0.778 | <0.001 | NA |
| Paired associates, delayed | -0.934 (-23.964, 22.096) | 0.697 | <0.001 | NA |
| CVLT, free recall | NA | NA | NA | NA |
| CVLT, cued recall | NA | NA | NA | NA |
| TMT-B | NA | NA | NA | NA |
| Digit span total | -1.233 (-17.114, 14.649) | 0.504 | <0.001 | NA |
| Digit span forward | 0.747 (0.603, 0.89) | **0.009** | <0.001 | NA |
| Digit span backward | 0.583 (-1.63, 2.797) | 0.461 | <0.001 | NA |
| FAS | NA | NA | NA | NA |
| **Spontaneous menopause** | | | | |
| MMSE | 0.137 (-0.558, 0.832) | 0.358 | <0.001 | NA |
| Paired associates, immediate | NA | NA | NA | NA |
| Paired associates, delayed | NA | NA | NA | NA |
| CVLT, free recall | -0.952 (-4.976, 3.072) | 0.260 | <0.001 | NA |
| CVLT, cued recall | 0.798 (-2.013, 3.609) | 0.229 | <0.001 | NA |
| TMT-B | -0.712 (-6.2, 4.777) | 0.350 | <0.001 | NA |
| Digit span total | -1.404 (-3.402, 0.593) | 0.072 | <0.001 | NA |
| Digit span forward | -0.676 (-3.244, 1.892) | 0.489 | <0.001 | NA |
| Digit span backward | 0.001 (-0.036, 0.037) | 0.851 | <0.001 | NA |
| FAS | 0.209 (-3.961, 4.379) | 0.706 | <0.001 | NA |
| **Effects of formulation** | | | | |
| **Estrogen-only** | | | | |
| MMSE | NA | NA | NA | NA |
| Paired associates, immediate | 1.196 (-3.857, 6.249) | 0.473 | <0.001 | NA |
| Paired associates, delayed | -0.528 (-3.238, 2.181) | 0.577 | <0.001 | NA |
| CVLT, free recall | -1.142 (-1.249, -1.036) | 0.005 | <0.001 | NA |
| CVLT, cued recall | -0.249 (-2.889, 2.391) | 0.741 | <0.001 | NA |
| TMT-B | 0.512 (-14.256, 15.28) | 0.740 | <0.001 | NA |
| Digit span total | -1.005 (-4.132, 2.122) | 0.337 | <0.001 | NA |
| Digit span forward | 0.747 (0.603, 0.89) | 0.009 | <0.001 | NA |
| Digit span backward | 0.583 (-1.63, 2.797) | 0.461 | <0.001 | NA |
| FAS | -1.325 (-2.962, 0.311) | 0.063 | <0.001 | NA |
| **Estrogen-progestogen** | | | | |
| MMSE | -1.853 (-2.974, -0.733) | 0.030 | <0.001 | NA |
| Paired associates, immediate | NA | NA | NA | NA |
| Paired associates, delayed | NA | NA | NA | NA |
| CVLT, free recall | 0.666 (-2.23, 3.561) | 0.231 | <0.001 | NA |
| CVLT, cued recall | 0.075 (-3.787, 3.938) | 0.845 | <0.001 | NA |
| TMT-B | NA | NA | NA | NA |
| Digit span total | -1.419 (-2.912, 0.073) | 0.053 | <0.001 | NA |
| Digit span forward | -0.775 (-4.143, 2.594) | 0.501 | <0.001 | NA |
| Digit span backward | 0.001 (-0.064, 0.067) | 0.840 | <0.001 | NA |
| FAS | 1.178 (-1.317, 3.673) | 0.112 | <0.001 | NA |

Standardized mean difference (SMD) and associated 95% confidence interval, CI, are reported for each cognitive test, along with corresponding p-values. Positive SMDs indicate an improvement in the treated group compared to the placebo group, whereas negative SMDs indicate the opposite effect. Analyses with fewer than 10 studies are marked as not applicable (NA) for the publication bias p-values.

Abbreviations: CVLT, California Verbal Learning Test; MMSE, Mini Mental State Examination; TMT-B, Trail Making Test B.

## Supplementary Table 5. Effects of MHT on individual cognitive tests by formulation and initiation timing

|  | **Standardized mean difference**  **(95% CI)** | **P-value** | **Heterogeneity**  **p-value** | **Publication**  **bias p-value** |
| --- | --- | --- | --- | --- |
| **Midlife estrogen-only treatment** | | | | |
| Paired associates, immediate | 2.249 (-8.144, 12.642) | 0.313 | <0.001 | NA |
| Paired associates, delayed | 0.267 (-1.105, 1.64) | 0.469 | <0.001 | NA |
| CVLT free recall | -0.972 (-7.566, 5.621) | 0.312 | <0.001 | NA |
| Digit span total | -2.450 (-6.478, 1.578) | 0.083 | <0.001 | NA |
| FAS | -1.982 (-4.206, 0.243) | 0.056 | <0.001 | NA |
| **Midlife estrogen-progestogen treatment** | | | | |
| NA | NA | NA | NA | NA |
| **Late-life estrogen-only treatment** | | | | |
| Digit span forward | 0.750 (0.624, 0.877) | 0.008 | <0.001 | NA |
| Digit span backward | -0.016 (-16.859, 16.828) | 0.992 | <0.001 | NA |
| FAS | -0.776 (-16.091, 14.539) | 0.636 | <0.001 | NA |
| **Late-life estrogen-progestogen treatment** | | | | |
| CVLT free recall | 0.696 (-2.014, 3.407) | 0.189 | <0.001 | NA |

Standardized mean difference (SMD) and associated 95% confidence interval, CI, are reported for each cognitive test, along with corresponding p-values. Positive SMDs indicate an improvement in the treated group compared to the placebo group, whereas negative SMDs indicate the opposite effect. Analyses with fewer than 10 studies are marked as not applicable (NA) for the publication bias p-values.

Abbreviations: CVLT, California Verbal Learning Test.

## Supplementary Table 6. Meta-regression analysis: MHT effects on cognitive domains and individual cognitive tests

|  | **Estimate** | **Lower 95% CI** | **Upper 95% CI** | **P-value** |
| --- | --- | --- | --- | --- |
| **Cognitive domains** | | | | |
| Intercept | 1.139 | -2.518 | 4.795 | 0.542 |
| Age at initiation: Late | 0.049 | -1.543 | 1.641 | 0.952 |
| Exposure: Estrogen-progesterone | -0.201 | -1.669 | 1.267 | 0.789 |
| Long duration of use: >1 year | -0.385 | -2.264 | 1.494 | 0.688 |
| Type of menopause: Surgical | -0.162 | -1.503 | 1.179 | 0.813 |
| Sample size: 100-500 | -0.483 | -2.647 | 1.680 | 0.661 |
| Sample size: 500+ | -0.488 | -1.668 | 0.693 | 0.418 |
| Domain: Verbal All | -0.693 | -4.019 | 2.632 | 0.683 |
| Domain: Visual | -1.273 | -4.712 | 2.166 | 0.468 |
| Domain: Visuospatial | 0.863 | -2.906 | 4.632 | 0.654 |
| Domain: Working memory | -0.703 | -4.067 | 2.660 | 0.682 |
| Domain: Fluency | 0.209 | -3.292 | 3.711 | 0.907 |
| Domain: Executive function | -3.803 | -7.628 | 0.022 | **0.051** |
| Domain: Language | 0.447 | -3.214 | 4.109 | 0.811 |
| **Individual cognitive tests** | | | | |
| Intercept | 2.138 | -4.672 | 8.948 | 0.538 |
| Age at initiation: Late | 3.341 | -2.491 | 9.173 | 0.262 |
| Exposure: Estrogen-progesterone | -0.653 | -5.135 | 3.829 | 0.775 |
| Long duration of use: >1 year | -4.205 | -11.084 | 2.674 | 0.231 |
| Type of menopause: Surgical | -0.113 | -4.284 | 4.058 | 0.958 |
| Sample size: 100-500 | -1.152 | -8.805 | 6.502 | 0.768 |
| Sample size: 500+ | -0.360 | -4.502 | 3.782 | 0.865 |
| Test: Paired associates immediate | -1.117 | -7.683 | 5.448 | 0.739 |
| Test: Paired associates delayed | -3.182 | -9.538 | 3.175 | 0.327 |
| Test: CVLT immediate | -1.749 | -7.725 | 4.227 | 0.566 |
| Test: CVLT delayed | -1.198 | -7.164 | 4.768 | 0.694 |
| Test: TMT-B | -0.027 | -17.015 | 16.962 | 0.998 |
| Test: Digit span total | -4.956 | -11.499 | 1.587 | 0.138 |
| Test: Digit span forward | -0.393 | -6.337 | 5.551 | 0.897 |
| Test: Digit span backwards | -0.724 | -6.669 | 5.221 | 0.811 |
| Test: FAS | -0.998 | -7.049 | 5.053 | 0.747 |

This table presents the results of meta-regression analyses assessing the impact of various factors on the effects of Menopausal Hormone Therapy (MHT) on cognitive domains and individual cognitive tests. The table includes the estimated effect sizes (estimate), their corresponding 95% confidence intervals (lower and upper 95% CI), and the associated P-values.

Abbreviations: CVLT, California Verbal Learning Test; TMT-B, Trail Making Test B.
